# Shear Stress‐Stimulated Trafficking of Endothelial Piezo1 to Splenic Capsule Boosts Spleen Contraction and Exercise Endurance

**DOI:** 10.1002/jev2.70375

**Published:** 2026-09-28

**Authors:** Jing Lou, Mingzong Yang, Yang Hu, Xuyun Liu, Han Li, Zihang Feng, Yuxin Xi, Changyang Xing, Lijun Yuan, Jia Li, Shengpeng Wang, Feng Gao, Xing Zhang

**Affiliations:** ^1^ Key Laboratory of Aerospace Medicine of the Ministry of Education, School of Aerospace Medicine Fourth Military Medical University Xi'an China; ^2^ Center for Mitochondrial Biology and Medicine, Key Laboratory of Biomedical Information Engineering of Ministry of Education, School of Life Science and Technology Xi'an Jiaotong University Xi'an China; ^3^ Department of Ultrasound Medicine, Tangdu Hospital Fourth Military Medical University Xi'an China; ^4^ State Key Laboratory of Cancer Biology, Department of Pathology, Xijing Hospital and School of Basic Medicine Fourth Military Medical University Xi'an China; ^5^ Department of Cardiovascular Medicine The First Affiliated Hospital of Xi'an Jiaotong University Xi'an China

**Keywords:** exercise, extracellular vesicles, Piezo1, shear stress, spleen contraction

## Abstract

Haemodynamic shear stress, sensed by vascular endothelial cells (ECs), is crucial for the functional integrity of ECs and the systemic benefits of exercise. Here, we observed that shear stress triggers the release of medium/large extracellular vesicles (m/lEVs), from ECs via activation of the mechanosensitive ion channel Piezo1. Endothelial Piezo1 activation induces Piezo1 translocation to the cell periphery, facilitating its trafficking from the vascular ECs to the smooth muscle cells in the splenic capsule. Within the splenic capsule, the translocated Piezo1 acts as a downstream effector of α1‐adrenergic receptor, boosting exercise‐induced spleen contraction and enhancing exercise endurance. Genetic or pharmacological inhibition of Piezo1 trafficking from ECs impaired spleen contraction during exercise and reduced exercise endurance in mice. Additionally, exercise‐induced m/lEV release contributes to safeguard endothelial function through modulating intracellular Piezo1 homeostasis. Furthermore, positive correlations between exercise‐induced increases in circulating Piezo1‐enriched m/lEVs and the degree of spleen contraction were also observed in human volunteers. These findings unveil a vasculo‐splenic axis during exercise, in which EC‐derived m/lEVs shuttle Piezo1 to maintain endothelial function and exercise endurance, highlighting a novel role of Piezo1 in inter‐organ communication.

## Graphical abstract

The vasculo‐splenic axis during exercise: shear stress triggers the release of medium/large extracellular vesicles from vascular endothelial cells via activation of Piezo1, shuttling Piezo1 to the smooth muscle cells in the splenic capsule; the translocated Piezo1 within the splenic capsule acts as a downstream effector of the α1‐adrenergic receptor, boosting exercise‐induced spleen contraction and enhancing exercise endurance.

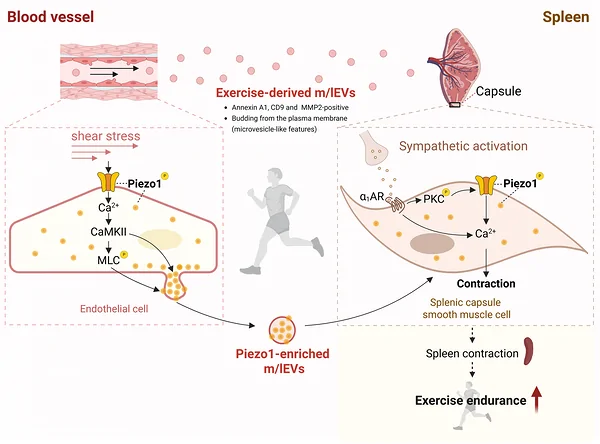

## 1 Introduction

The endothelium is a highly dynamic and interactive systemically disseminated organ with diverse functions. Its dysfunction is critically involved in almost every major life‐threatening and chronic human diseases (Augustin and Koh 2024). Emerging evidence has shown that the functional integrity of vascular endothelial cells (ECs) is critically dependent on blood flow‐imposed mechanical forces, especially for shear stress (Baeyens et al. 2016). ECs convert mechanical forces into biochemical signals through a series of mechanosensory membrane channels, among which Piezo1 plays a critical role in sensing shear stress. Piezo1 is a Ca^2+^‐permeable non‐selective cationic channel discovered in 2010 (Coste et al. 2010), demonstrating a three‐bladed propeller‐like structure (Zhao et al. 2018; Saotome et al. 2018). It mediates mechanotransduction across a broad range of cell types, and is involved in various physiological and pathological processes, including development, metabolism, inflammation and cancer (Xiao 2024; Wang et al. 2025). Endothelial Piezo1 is reported to be involved in the regulation of blood and lymphatic vessel development (Li et al. 2014; Ranade et al. 2014; Nonomura et al. 2018), angiogenesis (Bartoli et al. 2022), vascular permeability (Wang et al. 2022), vascular tone and blood pressure (Rode et al. 2017; Wang et al. 2016). Its activation triggers Ca^2+^ influx, which in turn activates multiple essential signalling pathways including nitric oxide (NO) synthesis and Yes‐associated protein (Yap) nuclear translocation (Wang et al. 2025).

Exercise elevates cardiac output and blood flow, increasing shear stress on the vascular endothelium (Green et al. 2017; Feng et al. 2025). Such an increase in shear stress is supposed to be an initial signal which contributes to the exercise‐afforded benefits in endothelial function (Green et al. 2017). Piezo1 acts as a central molecular sensor for haemodynamic changes during exercise (Beech 2025). Exercise‐induced activation of Piezo1 enhances both vascular contraction and relaxation in response to vasoactive factors (Rode et al. 2017), and promotes angiogenesis (Bartoli et al. 2022). Additionally, the altered blood flow further induces crosstalk between ECs and other types of cells, thus orchestrating the broad systemic benefits of exercise, partially through extracellular vesicles (EVs). Our previous study showed that shear stress induces EVs release from ECs, and these EVs mediate exercise‐induced benefits to the heart (Hou et al. 2019). In fact, shear stress is widely used to yield EVs in the field of biomedical engineering (Patel et al. 2019; Huang et al. 2025). EVs function as key mediators of intercellular communication through the transfer of bioactive cargo between cells, rewiring pathways and altering biological processes in both donor and recipient cells. EVs are classified based on their biogenesis mechanism (e.g., exosome, microvesicle and apoptotic bodies), concept (e.g., migrasome and oncosomes) and size (e.g., small EV, medium EV and large EV). Among these types of EVs, microvesicles (>200 nm in diameter) are medium/large EVs (m/lEVs) and formed through direct outward budding and shedding from the plasma membrane (Buzas 2023). Microvesicle biogenesis mechanism confers a high degree of cargo selectivity, enabling preferential enrichment of proteins located at or near the plasma membrane (Wang and Lu 2017; Xu et al. 2025). While the role of exosomes in exercise‐induced benefits has been well documented, the biological functions and mechanisms specific to microvesicle during exercise remain poorly understood.

In this study, we observed that Piezo1 activation by shear stress induces the release of m/lEVs with microvesicle‐like features from ECs both *in vitro* and *in vivo*. Specifically, these m/lEVs are characterized by an enrichment of Piezo1 protein in them, mediating Piezo1 trafficking from ECs to the splenic capsule to maintain both vascular function and exercise endurance. These findings illustrate a vasculo‐splenic axis during exercise, and highlight a critical role of EC‐derived m/lEVs as a vehicle of Piezo1 in inter‐organ communication.

## 2 Materials and Methods

Below describes the most relevant methods, and other methods are described in Supporting Information.

### 2.1 Human Study

A total of 20 healthy volunteers (13 males and 7 females), aged 22–35 years, were enrolled in this study (Table S1). All participants provided written informed consent prior to participation. The study protocol was approved by the Ethics Committee of Tangdu Hospital, the Second Affiliated Hospital of Fourth Military Medical University (Approval No. 202409‐08). Strict inclusion and exclusion criteria were applied to ensure the homogeneity of the cohort. Adults who regularly conducted aerobic exercise (at least 150 min of total time per week at an intensity of ∼60% of maximum heart rate) were eligible for enrolment to limit acute stress response during exercise. Exclusion criteria included a history of chronic metabolic diseases, cardiovascular disorders, hepatic or renal dysfunction, and recent use of medications affecting the parameters under investigation. To ensure the standardisation and safety of the human trial, all experimental procedures were strictly controlled. Prior to the exercise bout, all volunteers were instructed to fast for at least 8 h overnight and to abstain from caffeine, alcohol and strenuous physical activity for 24 h. They were also advised to maintain their regular sleep patterns and avoid any excessive eating or drinking. The acute exercise intervention was conducted in a dedicated climate‐controlled fitness room between 9:00 and 11:00 a.m. All volunteers underwent a single bout of acute treadmill running for 20 min. The treadmill was set at 0° inclination, with a running speed of 12 km/h for male volunteers and 8 km/h for female volunteers. Blood samples were collected and ultrasound assessments were performed both before and immediately after the exercise training.

Human splenic, vascular, and cardiac ultrasonography was systematically performed using a Philips Affiniti 50 system (Philips Healthcare, USA) with dedicated transducers for each anatomical target. All image acquisitions and measurements were performed by sonographers who were blinded to the participants. Spleen size was quantified in the coronal and transverse planes using a C6‐2 convex array transducer during breath‐hold to minimize respiratory motion artefact. For popliteal artery hemodynamic, an L12‐5 high‐frequency linear array transducer was employed. The arterial diameter was measured in B‐mode from longitudinal views, and pulsed‐wave Doppler with angle correction ≤60° was used to obtain the time‐averaged mean velocity (TAMV). Blood flow volume was calculated using the formula: Flow = π × (D/2)^2^ × TAMV, where D represents the vessel diameter. Cardiac output (CO) was assessed using an S4‐2 phased array transducer via pulsed‐wave Doppler interrogation at the left ventricular outflow tract (LVOT), where velocity‐time integral (VTI) and LVOT diameter were measured for stroke volume (SV) calculation (CO = SV × heart rate). Acoustic coupling was optimized with ultrasound gel, and participant positioning was standardized.

The following commercially available ELISA kits were used to measure protein levels in plasma of human: Human CD9 (Cusabio, China) and Human Piezo1 Elisa kit (Finetest, China). The manufacturer's instructions were followed.

### 2.2 Animals

All animal studies were performed in accordance with national guidelines and were approved by the Fourth Military Medical University Animal Care and Use Committee, Xi'an, China. C57BL/6 mice (male) at the age of 8 weeks were purchased from the Experimental Animal Centre of the Fourth Military Medical University (Xi'an, China). Endothelial‐specific (Piezo1_EC_^−/−^) and smooth muscle‐specific (Piezo1_SMC_^−/−^) Piezo1 knockout (KO) mice were constructed as previously reported (Wang et al. 2016). To generate liver‐specific Piezo1 KO mice (Piezo1_Hep_^−/−^), Piezo1^f/f^ mice were crossed with Alb‐Cre mice. Piezo1^f/f^ mice were crossed with SM22‐Cre mice to generate Piezo1_SMC_^−/−^ mice. Piezo1^f/f^ mice were crossed with Tek‐CreERT2 mice to generate Piezo1_EC_^−/−^ mice. EC‐specific knockout of Piezo1 was induced by injecting 6‐week‐old Piezo1^f/f^ Cre^+^ mice with tamoxifen (40 mg/kg/d for 10 days; i.p.). Tamoxifen‐injected Piezo1^f/f^ Cre^−^ mice were used as controls. Mice were used for experiments at 2 weeks after tamoxifen administration. Animals expressing the lacZ gene under the control of the Piezo1 promoter were obtained from the Knockout Mouse Project (KOMP) as described previously (Wang et al. 2020). Mice were fed standard chow and housed under a 12 h light/12 h dark cycle. For euthanasia, mice were anaesthetized with an intraperitoneal injection of sodium pentobarbital (100 mg/kg body weight, P‐010; Merck, Germany) and then euthanized via exsanguination.

### 2.3 Exercise Training and Treadmill Endurance Test

For the exercise model, mice were acclimatized to the treadmill system (ZH‐PT/5S, Anhui Zhenghua Biologic Apparatus Facilities, China) for 3 consecutive days prior to the exercise training. For acclimatisation, mice were subjected to exercise at 9 m/min for 20 min at 0° inclination, 10 m/min for 30 min at 0° inclination and 15 m/min for 40 min at 0° inclination on 3 different days. Mice were subjected to either a single bout of acute exercise or a one‐month chronic training (five times per week), with both protocols consisting of treadmill running at 18 m/min and 0° inclination for 1 h per training. Pantethine or Y27632 was administrated to exercised mice to block m/lEV release during exercise. Pantethine (150 mg/kg, HY‐B1028, MCE, USA) or Y27632 (5 mg/kg, HY‐10071, MCE, USA) was dissolved in saline and injected intraperitoneally at 30 min before each exercise. Injection of saline to a comparable volume (200 µL) as controls. For the endurance test, the initial speed was set at 13 m/min for 5 min at 0°inclination, followed by incremental increases of 1 m/min every minute until reaching 25 m/min, which was then maintained. The time and distance until exhaustion were recorded.

### 2.4 Shear Stress Induction In Vitro

For imaging experiments, human umbilical vein endothelial cells (HUVECs) were plated on μ‐slide I 0.4 Luers (Ibidi, Germany) until the cells reached 70%–80% confluency. The flow chamber was perfused with a constant flow rate using the computer‐controlled air pressure pump (Ibidi, Germany). HUVECs were exposed to laminar shear stress (LSS) at 0, 5, 15, 25 dyn/cm^2^ for 5 min, unless otherwise indicated.

For Western blot experiments to characterize EVs, HUVECs were seeded in 6‐well plates. The medium was replaced with 3 mL of fresh culture medium per well before transferring the plates to an orbital shaker (MT‐201‐BX, Labnique, USA). The orbital shaker was placed in a cell culture incubator to apply shear stress to HUVECs. The shear stress magnitude was estimated using the extended solution to Stokes’ second problem (Yan et al. 2025): $\mathrm{shear}\;\mathrm{stress}=\alpha \sqrt{\rho \eta {\left(2\pi f\right)}^{3}}$, where a is the radius of orbital rotation (1.1 cm), *ρ* is the density of the medium (1.0 g/mL), *η* is the viscosity of the medium (0.008 poise) and *f* is the frequency of rotation (rotations/second). Using this formula, a shear stress of 15 dyn/cm^2^ was achieved at a rotation frequency of 270 rpm. HUVECs from the same passage, not subjected to shear stress, were also plated in 6‐well plates and cultured for the same duration in a cell culture incubator. HUVECs were expose to LSS for 24 h to collect enough EVs.

### 2.5 m/lEV Isolation

Cell culture media (18 mL per plate) and blood samples were collected, and m/lEVs were isolated via sequential centrifugation and filtration according to a modified protocol as described previously (Ma et al. 2023; Kongsomros et al. 2022; Nieuwland and Siljander 2024). For blood samples, mice were fasted prior to blood collection to prevent initial lipoprotein contamination. Blood samples were avoided to expose to low temperature to minimize platelet activation and the release of platelet‐derived vesicles. Cell culture media were first centrifuged at 800 *g* for 10 min at 4°C to remove cells, and blood samples were centrifuged at 1200 *g* for 15 min at room temperature to isolate plasma. To optimize the method, plasma samples were diluted two‐fold with PBS (phosphate buffered saline) to reduce viscosity and improve separation efficiency. Samples were then centrifuged at 2000 *g* for 15 min at 4°C to pellet larger cell debris and remove remaining platelets. The supernatant was subsequently filtered with 5 µm filter in some of the experiments (EV‐related data in Figures 2 and 7). As we found that this filtration step is not necessary and does not affect the size, morphology, Piezo1 content and biological function of the isolated m/lEVs (Figure S1). The filtration process is omitted in other experiments. The samples were then centrifuged at 16,000 *g* for 30 min at 4°C to pellet m/lEVs. The resulting m/lEV pellet was washed with PBS and centrifuged again under the same conditions. Isolated m/lEVs were resuspended in PBS. For plasma samples, the resuspended m/lEVs were further purified using a 70 nm qEV size exclusion chromatography column (Izon Science, Christchurch, New Zealand). Consistent with standard size‐exclusion chromatography (SEC)‐based EV isolation procedures, the column was eluted with PBS, and a total of 12 sequential fractions (500 µL per fraction) were collected. The early fractions corresponding to the void volume were discarded, and fractions 7–9, which contained the highly enriched m/lEVs, were pooled and concentrated for downstream analysis. m/lEVs were isolated from equal numbers of donor cells or equal volumes of plasma.

### 2.6 Multi‐SIM Super‐Resolution Microscope

Single Slice‐SIM images of HUVECs cells were acquired on a Multi‐SIM imaging system (Beijing Nanolnsights‐tech Co. Ltd, China) with a 100X/1.49 NA oil objective (Nikon, Japan), semiconductor lasers (405 nm)/solid‐state lasers (488, 561 and 640 nm) and a scientific Complementary Metal‐Oxide‐Semiconductor (sCMOS) camera (Photometrics Kinetix, China). To obtain optimal images, immersion oils with refractive indices of 1.518 were used for HUVECs cells on glass coverslips. The microscope is routinely calibrated with 100 nm fluorescent spheres to calculate both the lateral and axial limits of image resolution. SIM image stacks were reconstructed using SI‐Recon 2.23.3 (Beijing Nanolnsights‐tech Co. Ltd, China) with the following settings: pixel size 30.6 nm; channel‐specific optical transfer functions; Wiener filter constant 0.01 for 2D mode; discard Negative Intensities background. Then the reconstructed SIM image was denoised with total variation constraint. Pixel registration was corrected to be less than 1 pixel for all channels using 100 nm fluorescence beads.

### 2.7 Confocal Imaging

The membranes of EVs and cells were fluorescently labelled using the lipophilic dye PKH26 (5 µM, Sigma–Aldrich, USA), following the manufacturer's instructions. PKH26 fluorescence was visualized on a Zeiss LSM 800 confocal microscope (Germany) using the 561/570–620 nm channel. For the analysis of the percentage of cells releasing EVs, HUVECs were exposed to an LSS of 15 dyn/cm^2^ for 5 min, with the entire process recorded by confocal microscopy. The confocal time series settings consisted of 60 cycles, with an interval of 5 s between each cycle. Cells were counted as EV‐releasing only when clear release of EVs from the parent cell was observed. If the origin of the EVs could not be determined, the cells were excluded from the count. Similarly, when analysing the number of m/lEVs per cell, only those EVs definitively released from the cell were counted, ensuring that free PKH26 micelles were not mistakenly quantified as EVs. Cells were loaded with Fluo‐4 AM (10 µM, Thermo Fisher Scientific, USA) at 37°C for 10 min to image Ca^2+^. Intracellular Ca^2+^ was imaged on a Zeiss LSM 800 confocal microscope (Germany) using the 488/500–550 nm channel. Fluorescence intensities of images were analyzed using ImageJ Fiji (NIH, USA).

### 2.8 Particle Size Characterisation

The particle size and distribution were characterized using nanoparticle tracking analysis (NTA) and dynamic light scattering (DLS). For single‐particle tracking, NTA was performed by a NanoSight NS300 (Malvern, UK) equipped with a 405 nm laser and a sCMOS camera. Samples diluted 100‐fold in filtered PBS were captured for 60 s (camera level 14, detection threshold 3), and analyzed via NTA 3.2 software. The image of filtered PBS was taken to verify that the diluent had no particle in it. The NTA 3.2 Dev Build 3.2.16 software (Malvern, UK) was used for capturing and data analysis. The bulk hydrodynamic diameter (Z‐average) and polydispersity index (PDI) were measured by DLS using a Zetasizer Nano ZS (Malvern, UK) with a 633 nm laser at a 173° scattering angle. DLS measurements were conducted in triplicate at 25°C after a 120 s equilibration.

### 2.9 Transmission Electron Microscope (TEM)

To evaluate the morphology of isolated m/lEVs, negative staining was performed. Briefly, m/lEV suspensions were deposited onto formvar/carbon‐coated copper grids, negatively stained with 2% phosphotungstic acid, and imaged using the TEM (HT7800, HITACHI, Japan). For observation of cellular budding structures, HUVECs were fixed with 3% glutaraldehyde and post‐fixed in 1% osmium tetroxide, followed by acetone dehydration and Epon 812 embedding. Semi‐thin sections were toluidine blue‐stained for orientation. Ultrathin sections were prepared with a diamond knife, stained with uranyl acetate and lead citrate, and imaged using the TEM (JEM‐1400FLASH, JEOL Ltd., Japan). For immuno‐electron microscopy, samples were fixed in 3% glutaraldehyde, dehydrated in acetone, and resin‐embedded. Ultrathin sections on nickel grids were etched with 3% H_2_O_2_, rinsed, and blocked with serum. Grids were incubated with primary antibody (Piezo1, 15939, Proteintech, China) at 4°C, followed by colloidal gold‐conjugated secondary antibody. After washing and counterstaining with uranyl acetate and lead citrate, samples were imaged with the TEM (JEM‐1400FLASH, JEOL Ltd., Japan).

### 2.10 Scanning Electron Microscope (SEM)

To visualize the 3D topography of vesicular surface budding, HUVECs seeded on sterile coverslips were primarily fixed with 3% glutaraldehyde for 2 h at room temperature. The samples were post‐fixed in 1% osmium tetroxide for 2 h at room temperature, dehydrated through a graded ethanol series, transferred to isoamyl acetate, and dried using a critical point dryer. After drying, samples were mounted on metallic stubs with carbon adhesive tabs, sputter‐coated with gold, and imaged using a scanning electron microscope (Quattro S, Thermo Fisher Scientific, USA).

### 2.11 Ultrasonic Examination

High‐resolution ultrasound imaging of the spleen and measurement of heart‐carotid pulse wave velocity in mice were performed using a Vevo 2100 system (VisualSonics Inc., Canada). Prior to the examinations, all mice were anesthetized via inhalation of 1%–2% isoflurane in oxygen. Core body temperature was maintained at 36°C–37°C using a heated platform throughout the procedure. For mice spleen ultrasonic imaging, each mouse was positioned in right lateral recumbency. Fur on the left abdominal area was chemically depilated, and ultrasound transmission gel was applied to ensure adequate acoustic coupling. The spleen was identified in the left upper quadrant, beneath the rib cage. Static B‐mode images were captured in the coronal plane to assess splenic morphology and echogenicity, and spleen size was measured using the machine's onboard caliper tools. For pulse wave velocity assessment, flow pressure waveforms generated by cardiac activity and arterial pulses were captured. The pulse wave velocity was then calculated as the ratio of the travelled distance between measurement sites to the transit time.

### 2.12 Splenic Denervation

Surgical denervation of the spleen was performed following a previously published protocol (Zhang et al. 2020). Three‐week‐old male mice were anaesthetized via an intraperitoneal injection of sodium pentobarbital (70 mg/kg body weight; Merck). Following hair removal in mice, the abdominal cavity was accessed through a midline incision. The spleen was carefully separated from the peritoneal cavity and exposing the primary vascular supply structures. Absolute ethanol was repeatedly applied with cotton tips to the vascular trees for 5–10 s each time, at 5 s intervals, and about seven times in total in order to deplete the splenic nerve fibres that run along them. To prevent vasospasms and subsequent vascular damage or splenic necrosis, ethanol application was carefully controlled. Sham‐group mice underwent the identical surgical operation, with normal saline replacing absolute ethanol. Mice were monitored postoperatively for 5 weeks before exercise and splenic ultrasound experiments. Splenic denervation efficacy was assessed one week postoperatively.

### 2.13 Splenic Capsule Smooth Muscle Cells (scSMCs) Isolation and Culture

scSMCs were isolated from adult C57BL/6 mice. The spleen was removed from the mouse, and the splenic capsule (a translucent membrane) was carefully dissected and cut into small fragments. The tissue was digested using 1 mg/mL collagenase type II (MCE, USA), 0.744 units/mL elastase (MCE, USA), containing 1% penicillin and streptomycin (Sigma–Aldrich, USA). The digested splenic capsule samples were passed through 100 and 70 µm cell filters in turn to remove undigested tissue. The cell suspensions were then centrifuged at 400 *g* for 5 min. Pelleted cells were resuspended and subsequently cultured in Dulbecco's Modified Eagle Medium (DMEM, Sigma–Aldrich, USA), containing 10% foetal bovine serum (FBS, Gibco, USA), 100 U/mL penicillin and 100 µg/mL streptomycin. The purity of SMCs was confirmed through immunofluorescence staining for α‐smooth muscle actin (α‐SMA, 1:200, 48938S, CST, USA) (>70%).

### 2.14 scSMCs Contraction Assay

A collagen gel contraction assay was performed to evaluate cellular contractility. Briefly, type I collagen rat tail (Corning, USA) was mixed with 10× PBS and neutralized with NaOH according to the manufacturer's instructions. Cells were resuspended in the collagen solution at a density of 2 × 10^5^ cells/mL and seeded in 24‐well plates. After polymerisation at 37°C for 1 h, the gels were gently released from the well walls and overlaid with culture medium. Contraction was monitored over 24 h, and gel areas were quantified using ImageJ software (NIH, USA). The degree of contraction was expressed as the percentage reduction in gel area relative to the initial size.

### 2.15 HUVECs Culture

HUVECs were obtained from Procell Life Science & Technology Co., Ltd. (China). Cells were cultured in endothelial cell medium containing 5% FBS, 1% EC growth supplement and 1% penicillin/streptomycin solution (1001, ScienCell, USA). To minimize contamination from serum‐derived EVs, the FBS used in the culture medium was pre‐cleared by ultracentrifugation at 100,000 × *g* before use. The cells were maintained for no more than eight passages. Routine PCR‐based mycoplasma screening was performed every two weeks on all cell cultures used in this study, and no positive results were obtained throughout the experimental period.

### 2.16 Rotarod Test

Rotarod coordination and fatigue were assessed using a 5‐lane rotarod system (JLBehvRRTG; Shanghai Jiliang Software Technology, China). In brief, the mice were acclimated to the rotarod with three trials every day for a period of 3 days. For coordination assay, the rotarod accelerated from 4 revolutions per minute (rpm) to 40 rpm in 5 min and maintained the maximum speed until the mice fell. Latency to fall was recorded for each trial. After 3 days’ rest, mice underwent a rotarod fatigue assay. The rotarod speed started at 15 rpm for 20 min and accelerated to 22 rpm in 16 min, then the maximum speed of 22 rpm was used for the final time of 1 h. Falls were immediately recorded, and mice were promptly returned to the rod. The number of cumulative falls binned every 5 min was recorded for each animal.

### 2.17 Blood Pressure Measurement

Mice were placed in animal holders on a far‐infrared warming platform to maintain their body temperature at 37°C. The blood pressure of conscious mice was measured using a volume pressure recording sensor system (BP‐2010A, Softron, Japan). After allowing time for stabilisation, systolic blood pressure was carefully monitored.

### 2.18 Measurement of Vascular Function

Mice were euthanized and the descending aorta was carefully excised and placed in ice‐cold physiological saline solution. The contractile force was recorded using a PowerLab Chart v 7.2.1 program (model 610 M, Danish Myo Technology, Denmark). Aortic rings were precontracted with phenylephrine (PE, 10^−3^ M, Sigma–Aldrich, USA). Endothelium‐dependent vasorelaxation was evoked by acetylcholine (ACh, 10^−9^ to 10^−4^ M, Sigma–Aldrich, USA).

### 2.19 Statistical Analyses

Values are presented as mean ± standard deviation (SD) for human data and as mean ± standard error of the mean (SEM) for murine experimental data. Data normality was evaluated using the Shapiro–Wilk test. For comparisons between two groups with normal distributions, an unpaired Student's *t*‐test was employed. Otherwise, the non‐parametric Mann–Whitney *U* test was utilized. To compare between multiple groups, One‐way ANOVA followed by Tukey's multiple comparison test and two‐way ANOVA followed by Bonferroni's multiple comparison test were used. Pearson correlation coefficient and multiple linear regression analyses were adopted to investigate the associations between spleen contraction and other factors. Values of *p *< 0.05 were considered statistically significant.

### 2.20 Machine Learning Analysis

Statistical analysis and machine learning were performed in Python 3.9 using pandas (v1.3.5), scikit‐learn (v1.0.2) and SciPy (v1.7.0). Clinical data were extracted from Table S2. Pearson correlations between two spleen contraction targets and eight predictor ratios were computed and visualized via clustered heatmaps. The dataset was split 70:30 for training and testing. A unified preprocessing pipeline standardized continuous features. Four regression models were evaluated via 5‐fold cross‐validation, with the optimal model selected based on the lowest cross‐validated Mean Squared Error (MSE). Final models were assessed using MSE and R^2^. Feature importance was interpreted using Gini importance for tree‐based models and standardized coefficients for linear models. All stochastic processes were seeded (random_state = 42) to ensure reproducibility, and results were programmatically archived.

## 3 Results

### 3.1 LSS Boosts m/lEV Release From ECs Through Activation of Piezo1

Upon LSS (15 dyn/cm^2^) stimulation, lots of EVs were released from HUVECs labelled with PKH26, a lipophilic membrane dye, in a time‐ and dose‐dependent manner (Figure 1A‐C; Video S1), without affecting the cell viability (Figure S2A). These EVs were circular in shape with an intact membrane structure, and formed via plasma membrane budding. Without LSS (0 dyn/cm^2^), only 15.33% of ECs released EVs, and LSS (15 dyn/cm^2^ for 300 s) increased the rate of EV‐releasing cells to 48.83% (Figure 1D). LSS increased the average EV‐releasing number per cell from 0.52 to 2.73 (Figure 1D). Super‐resolution optical imaging and transmission electron microscopy imaging showed that these EVs were formed via plasma membrane budding (Figure 1E, F). Consistent with these findings, scanning electron microscopy of partially attached cells facilitated the observation of membrane‐continuous budding necks, illustrating the dynamic process of EV release (Figure 1G). Additionally, these EVs were Annexin A1‐positive (a microvesicle biomarker) (Figure 1H). Based on these observations across multiple imaging methods, the LSS‐induced EVs exhibit a relatively large size and are characterized by outward budding from the plasma membrane. Therefore, in accordance with the MISEV guidelines (Welsh et al. 2024), we classify these LSS‐induced EVs as m/lEVs with microvesicle‐like features. Next, we isolated these m/lEVs through differential centrifugation (Figure 1I). The morphology of the isolated m/lEVs was characterized using transmission electron microscopy (Figure 1J). Subsequently, particle size was determined by NTA and DLS. NTA analysis showed comparable median particle sizes of 331.05 and 325.35 nm in the two groups (Figure 1K). DLS analysis showed mean hydrodynamic diameters of 562.30 nm with a polydispersity index of 0.16 in the control group and 613.00 nm with a polydispersity index of 0.25 in the LSS group (Figure S2B). The results of DLS displayed a larger size of the isolated EVs due to its determination of the intensity‐weighted hydrodynamic diameter, which is more sensitive to larger particles (Vogel et al. 2021). NTA analysis revealed that LSS increased the concentration of m/lEVs (Figure 1L). LSS increased the contents of microvesicle markers, including Annexin A1, CD9, and matrix metalloproteinase 2 (MMP2), as well as the EV markers TSG101 and ALIX, in the m/lEV fraction (Jeppesen et al. 2019; Hou et al. 2020; Mathieu et al. 2021), while the endoplasmic reticulum marker Calnexin, the lipoprotein marker APOB, and the apoptotic body marker cleaved Caspase‐3 were undetected (Figure 1M). To test whether Piezo1 was involved in this process, ECs were directly treated with Yoda1 (Piezo1 agonist, 2 µM). Yoda1 increased m/lEV release from ECs as detected by both western blot of isolated m/lEVs and confocal imaging of living cells (Figure 1N, O). Conversely, GsMTx4 (Piezo1 inhibitor, 5 µM) blocked the effect of LSS on m/lEV release (Figure 1P). Previous studies established that LSS activates Piezo1 through phosphorylation of Piezo1 at Ser1612 (S1612) (Zhang et al. 2024). Consistently, we observed that LSS induced Piezo1 phosphorylation in ECs (Figure 1Q), and LSS failed to increase m/lEV release in cells with Piezo1 reduction by siRNA (Figure 1R). Other types of cells, including vascular smooth muscle cells (VSMCs), A549 cells, HepG2 cells and human aortic endothelial cells (HAECs), also released m/lEVs following Yoda1 stimulation (Figure S2C). DLS analysis showed that LSS did not alter the mean hydrodynamic diameter of the released m/lEVs (Figure S2D). These findings indicate that LSS triggers the release of m/lEVs with microvesicle‐like features from ECs through activation of Piezo1.

**FIGURE 1:**
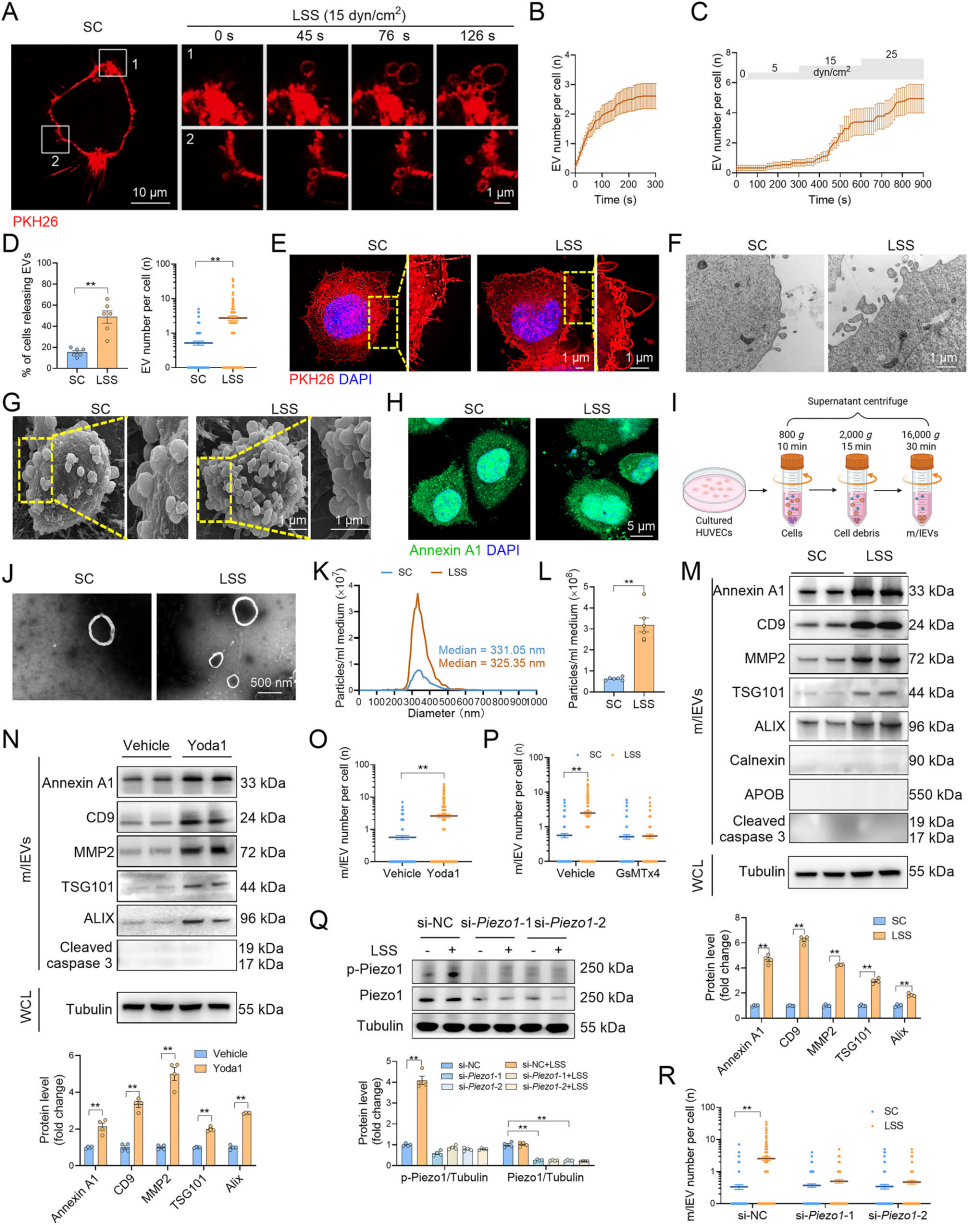
Laminar shear stress (LSS) boosts medium/large extracellular vesicle (m/lEV) release from HUVECs through activation of Piezo1. (A) In contrast to the static control (SC), LSS (15 dyn/cm^2^) stimulated substantial EV release from HUVECs as visualized by a confocal microscope (*n* = 10 cells from five independent experiments). PKH26 was used to label the membrane structures of EVs and cells. (B) Cumulative EV‐releasing number per cell along with time in response to a single dose of LSS (*n* = 100 cells from 10 independent experiments). (C) Cumulative EV‐releasing number per cell along with time in response to increasing doses of LSS (*n* = 18 cells from three independent experiments). (D) Quantification of percentage of cells releasing EVs and EV‐releasing number per cell. Left panel: the percentage of cells releasing EVs in total cells (*n* = 6). Right panel: the EV‐releasing number per cell (*n* = 200 cells from six independent experiments). (E) Single Slice‐SIM imaging revealed that LSS enhanced EV release through membrane budding (*n* = 50 cells from three independent experiments). (F) Transmissible electron microscope imaging demonstrated that LSS triggered the release of EVs shed from the membrane of HUVECs (*n* = 20 cells from three independent experiments). (G) Scanning electron microscope imaging revealed that EVs were distributed on the cell surface, and some of them were budding from the cell surface with membrane‐continuous necks (*n* = 20 cells from three independent experiments). (H) LSS‐induced EVs were Annexin A1‐positive (microvesicle biomarker) (*n* = 3). (I) The protocol for isolation of m/lEVs from HUVECs. (J) The morphology of the isolated m/lEVs was imaged using transmission electron microscopy (*n* = 4). (K) LSS did not alter the size distribution of m/lEVs as detected by NTA. (L) Quantification of particle number of m/lEVs by NTA (*n* = 6). (M) Western blot analysis of LSS‐released m/lEVs with specific biomarkers (Annexin A1, CD9, MMP2, TSG101, ALIX, Calnexin, APOB and cleaved caspase 3). WCL: Whole cell lysate (*n* = 4). (N) Yoda1 (Piezo1 agonist, 2 µM) promoted m/lEV release as detected by Western blot (*n* = 4). (O) Yoda1 enhanced m/lEV release as visualized by confocal microscope (*n* = 200 cells from three independent experiments). (P) GsMTx4 (Piezo1 inhibitor, 5 µM) suppressed LSS‐induced m/lEV release as visualized by confocal microscope (*n* = 200 cells from three independent experiments). (Q) LSS elevated Piezo1 phosphorylation (*n* = 4). (R) Piezo1 reduction reduced LSS‐induced m/lEV release as visualized by confocal microscope (*n* = 200 cells from three independent experiments). In imaging experiments, HUVECs were subjected to either 15 dyn/cm^2^ LSS for 300 s or Yoda1 for 10 min, unless otherwise indicated. For Western blot analysis, HUVECs were treated with 15 dyn/cm^2^ LSS or Yoda1 for 24 h to ensure adequate EV collection. All inhibitors were added 5 min prior to LSS stimulation. Data are presented as mean ± SEM. **p < 0.01.

### 3.2 Piezo1 Activation Induces m/lEV Release Through Ca^2+^‐Calcium/Calmodulin Dependent Protein Kinase II (CaMKII)‐Myosin Light Chain (MLC) Signalling

Given the critical role of Piezo1 in mechanical sensing, its subcellular repositioning may constitute the initial step in signal amplification (Bertaccini et al. 2025). Therefore, we performed immunofluorescence analysis of Piezo1 following LSS (15 dyn/cm^2^) stimulation. Interestingly, LSS induced a translocation of Piezo1 from the cytoplasm to regions adjacent to the plasma membrane in ECs (Figure 2A). These Piezo1‐enriched areas at the cell periphery were colocalized with the plasma membrane budding sites (Figure 2A). Following Piezo1 reduction, the membrane‐oriented translocation of Piezo1 in response to LSS was markedly attenuated (Figure 2B). Ca^2+^ acts as a critical downstream signalling molecule of Piezo1. Application of thapsigargin (TG) (1 µM), a potent inhibitor of the sarcoplasmic reticulum calcium ATPase (SERCA) pump that increases cytosolic calcium levels but inhibit the Ca^2+^ fluctuation in the intracellular space, promoted Piezo1 translocation to the cell periphery at baseline, but inhibited the effect of Yoda1 (2 µM) on promoting Piezo1 translocation (Figure 2C). Meanwhile, chelation of intracellular Ca^2+^ with 1,2‐bis (o‐aminophenoxy)ethane‐N,N,N′,N′‐tetraacetic acid (BAPTA, 10 µM) abolished Piezo1 translocation to the cell periphery induced by Yoda1, suggesting a Ca^2+^ influx‐dependent manner in LSS‐induced Piezo1 translocation (Figure 2C). Additionally, both TG and BAPTA inhibited Yoda1‐induced m/lEV release (Figure 2D,E, Figure S3A), suggesting that LSS‐induced Piezo1 translocation and EV release are mediated by Piezo1 activation‐induced Ca^2+^ influx in intracellular space.

**FIGURE 2:**
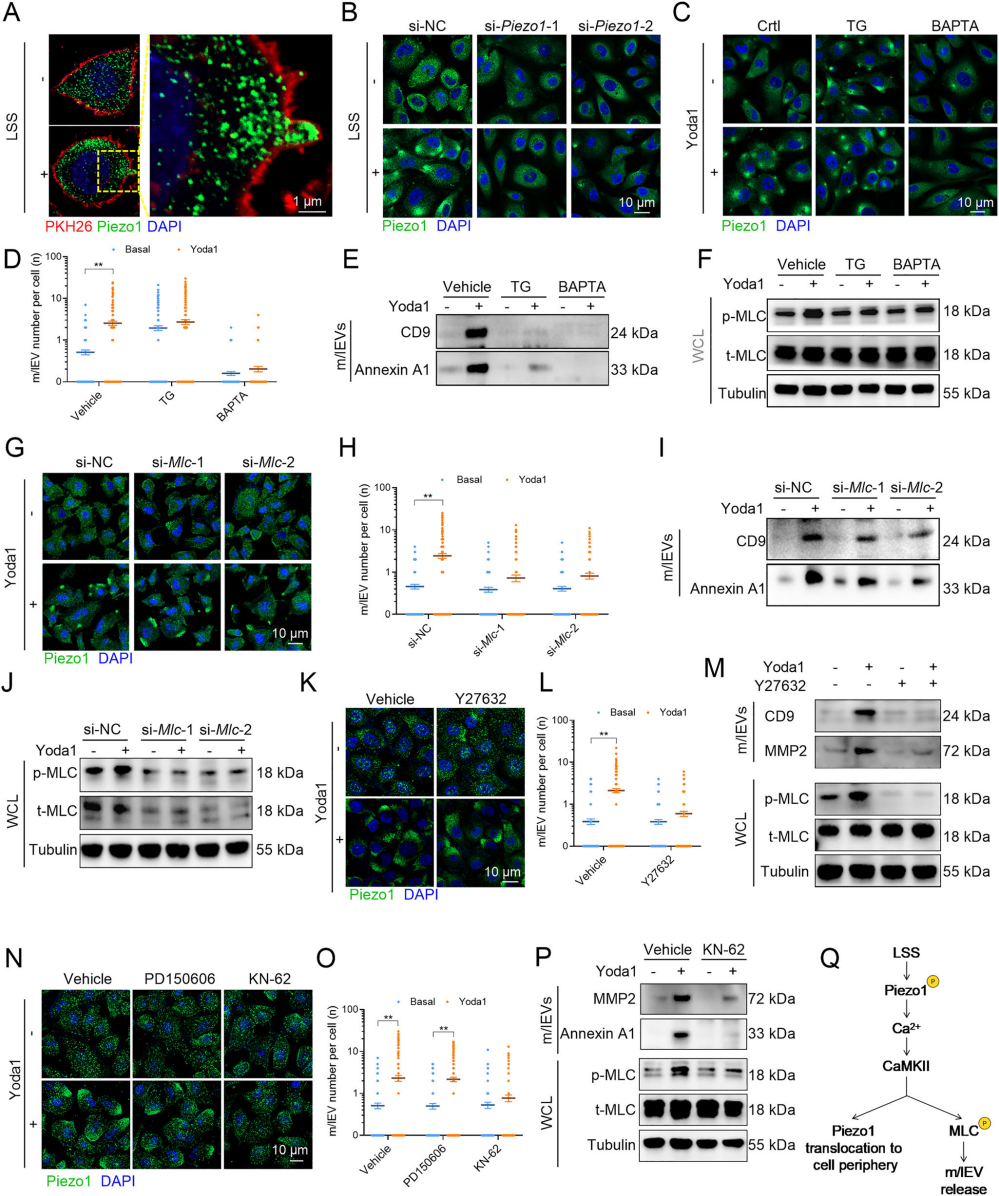
LSS triggers m/lEV release via Ca^2+^‐CaMKII‐MLC signalling. (A) LSS (15 dyn/cm^2^ for 300 s) induced Piezo1 translocation to the cell periphery colocalising with the microvesicle formation site (*n* = 3). PKH26, a lipophilic membrane dye for labelling the membrane structures of EVs and cells. (B) Piezo1 reduction by siRNA suppressed LSS‐induced Piezo1 translocation to the cell periphery (*n* = 3). (C) Inhibition of Ca^2+^ signal through Thapsigargin (TG, 1 µM) and BAPTA (10 µM) blocked Yoda1 (Piezo1 agonist, 2 µM)‐induced Piezo1 translocation to the cell periphery (*n* = 3). (D) TG and BAPTA inhibited Yoda1‐induced m/lEV release as imaged by confocal microscope (*n* = 200 cells from three independent experiments). (E) TG and BAPTA suppressed Yoda1‐induced m/lEV release as detected by Western blot (*n* = 3). (F) TG and BAPTA inhibited Yoda1‐induced MLC phosphorylation in HUVECs (*n* = 3). (G) MLC reduction did not affect the Yoda1‐induced Piezo1 translocation to the cell periphery (*n* = 3). (H) MLC reduction reduced Yoda1‐induced m/lEV release as visualized by confocal microscope (*n* = 200 cells from three independent experiments). (I) MLC reduction attenuated Yoda1‐induced m/lEV release as detected by Western blot (*n* = 3). (J) MLC reduction decreased Yoda1‐induced MLC phosphorylation (*n* = 3). (K) Pharmacological inhibition of MLC phosphorylation by Y27632 (2 µM) did not affect the Yoda1‐induced Piezo1 translocation to the cell periphery (*n* = 3). (L) Y27632 reduced Yoda1‐induced m/lEV release as visualized by confocal microscope (*n* = 200 cells from three independent experiments). (M) Y27632 attenuated Yoda1‐induced m/lEV release and MLC phosphorylation as detected by Western blot (*n* = 3). (N) KN‐62 (CaMKII inhibitor, 1 µM), but not PD150606 (Calpain inhibitor, 10 µM), blocked Yoda1‐induced Piezo1 translocation to the cell periphery (*n* = 3). (O) KN‐62 reduced Yoda1‐induced m/lEV release as imaged by confocal microscope (*n* = 200 cells from three independent experiments). (P) KN‐62 attenuated Yoda1‐induced m/lEV release and MLC phosphorylation as detected by Western blot (*n* = 3). (Q) Schematic diagram showing that Piezo1 activation‐induced Piezo1 translocation to the cell periphery and m/lEV release are mediated by Ca^2+^‐CaMKII signalling. In imaging experiments and subsequent quantitative analysis, HUVECs were subjected to either 15 dyn/cm^2^ LSS for 300 s or Yoda1 for 10 min. For Western blot analysis, HUVECs were treated with Yoda1 for 24 h to ensure adequate EV collection. The quantified results of the Western blot were shown in Figure S3. All inhibitors were added 5 min prior to Yoda1 stimulation. Data are presented as mean ± SEM. **p < 0.01.

Previous studies have demonstrated that MLC phosphorylation is required for the actomyosin contractility at the ‘necks’ of microvesicles and their subsequent abscission and shedding from the plasma membrane (Muralidharan‐Chari et al. 2009). TG and BAPTA inhibited Yoda1‐induced MLC phosphorylation (Figure 2F, Figure S3A). MLC reduction by siRNA did not affect Yoda1‐promoted Piezo1 translocation to the cell periphery, but inhibited Yoda1‐induced MLC phosphorylation and m/lEV release (Figure 2G–J, Figure S3B). Similarly, pharmacological inhibition of MLC phosphorylation with Y27632 (2 µM) also showed no effect on Yoda1‐induced Piezo1 translocation to the cell periphery, but reduced m/lEV release (Figure 2K–M, Figure S3C). These results suggest that MCL phosphorylation is required for LSS‐induced m/lEV release, but not for Piezo1 translocation. CaMKII and Calpain are two key upstream regulators that phosphorylate MLC, which facilitate cytoskeleton contraction and promote membrane budding and microvesicle formation (van Niel et al. 2018). Pharmacological inhibition of Calpain by PD150606 (10 µM) showed no effect on Yoda1‐induced Piezo1 translocation to the cell periphery and m/lEV release (Figure 2N,O). However, pharmacological inhibition of CaMKII by KN‐62 (1 µM) blocked Yoda1‐induced Piezo1 translocation to the cell periphery and m/lEV release (Figure 2N–P, Figure S3D). These results suggest that Piezo1 activation‐induced Piezo1 translocation to the cell periphery and m/lEV release are mediated by Ca^2+^‐CaMKII signalling, where MLC phosphorylation is required for m/lEV release but not Piezo1 translocation (Figure 2Q).

### 3.3 Inhibiting m/lEV Release Blocks the Benefits of LSS in ECs

Next, we examined the functional impact of m/lEV release on ECs. We used two different inhibitors to suppress the release of m/lEVs. Specifically, cells were treated with Y27632 (2 µM), a ROCK inhibitor that suppresses actin cytoskeletal reorganisation, and pantethine (50 µM) which impairs microvesicle shedding by blocking cholesterol synthesis and phosphatidylserine translocation (Kim et al. 2022). Treatment with Yoda1 (2 µM) for 24 h induced m/lEV release, whereas both pantethine and Y27632 inhibited Yoda1's effect on promotion of m/lEV release in ECs (Figure 3A). Yoda1 increased the capacity of endothelial angiogenesis, as evidenced by upregulated cell viability, promoted cell migration and tube formation, and increased phosphorylation of vascular endothelial growth receptor 2 (VEGFR2) (Figure 3B,C, Figure S4A,C). It also elevated NO production in response to acetylcholine (ACh, 10 µM) stimulation, and increased the phosphorylation of endothelial nitric oxide synthase (eNOS) (Figure S4B,C). Inhibition of m/lEV release by Y27632 or pantethine inhibited the beneficial effects induced by Yoda1 in ECs (Figure 3B,C, Figure S4A–C). These results suggest that promoting m/lEV release contributes to Yoda1‐induced benefits in ECs.

**FIGURE 3:**
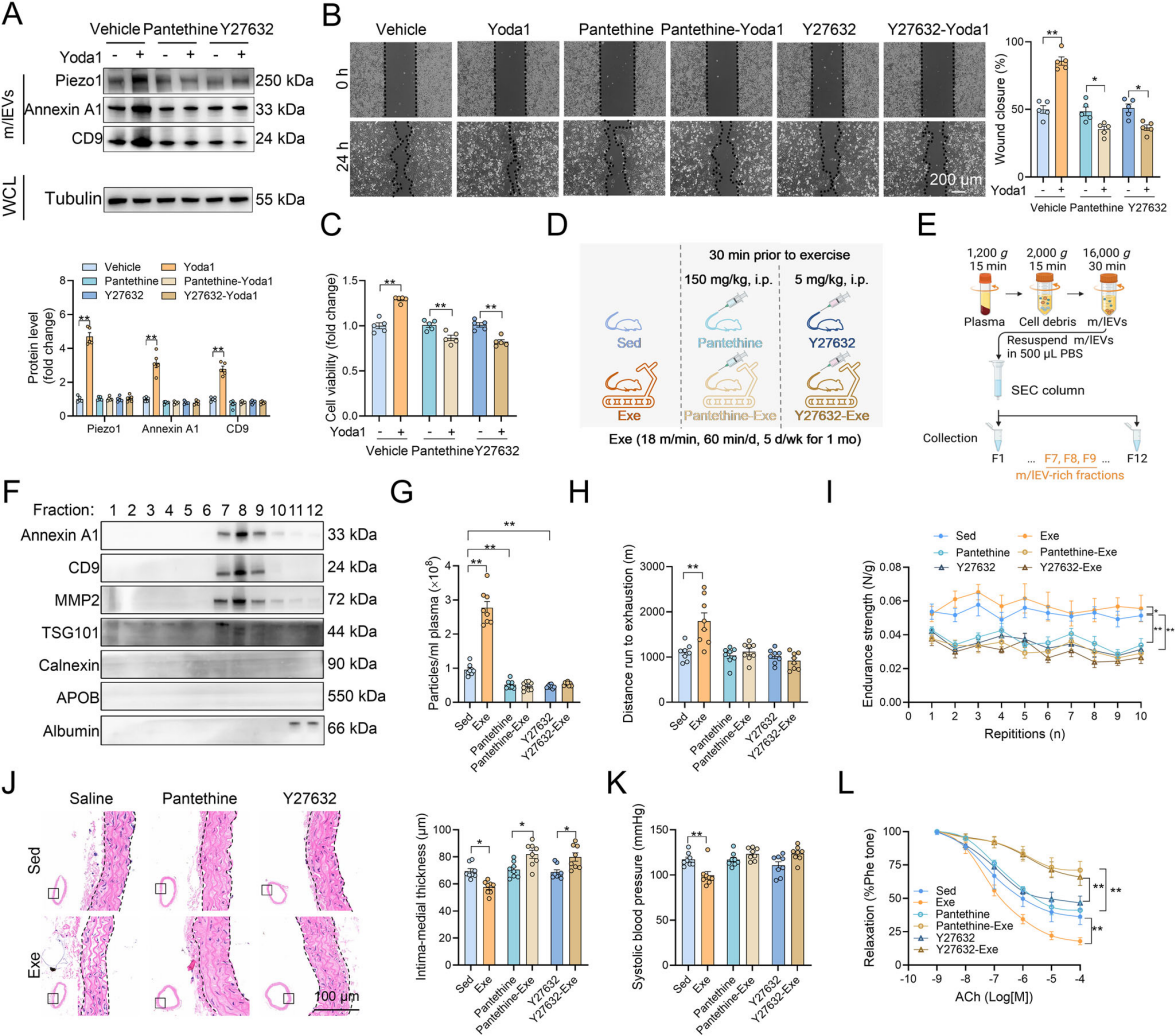
Inhibition of m/lEV release inhibits the effects of shear stress on promoting endothelial function. (A) Treatment with Yoda1 (Piezo1 agonist, 2 µM) for 24 h increased m/lEV release, whereas both pantethine and Y27632 attenuated this effect (*n* = 5). (B, C) Inhibition of m/lEV release by pantethine (50 µM) and Y27632 (2 µM) abolished Yoda1‐induced benefits in HUVECs, as determined by cell migration (B) and cell viability (C) (*n* = 5). HUVECs were pre‐treated with pantethine or Y27632 for 5 min followed by Yoda1 treatment for 24 h. (D) Pantethine (150 mg/kg, i.p.) and Y27632 (5 mg/kg, i.p.) were used to test whether inhibiting m/lEV release causes vascular endothelial dysfunction in vivo. Pantethine or Y27632 was injected to mice at 30 min prior to each exercise. The mice were subjected to treadmill exercise at 18 m/min, 60 min/d, 5 days a week, for 1 month. All indices were tested after 1 month of exercise. (E) Schematic diagram of plasma m/lEV isolation and purification. (F) The presence of the EV markers Annexin A1, CD9, MMP2 and TSG101 were determined in SEC fractions 7–9 (500 µL/fraction) using Western blotting. Fractions 7–9 were identified as the m/lEV‐rich fractions (*n* = 4). (G) Both pantethine and Y27632 blocked the upregulation of circulating m/lEVs induced by exercise, as determined by NTA (*n* = 8). (H) The exercise‐induced increase in exhaustion distance on the treadmill was abolished by pantethine and Y27632 (*n* = 8). (I) Pantethine or Y27632 suppressed exercise‐promoted endurance of forelimb grip strength (*n* = 6). (J) Pantethine and Y27632 reduced the effect of exercise on decreasing aortic intima‐media thickness (*n* = 8). (K) Treatment with pantethine or Y27632 blocked the effect of exercise on reducing blood pressure (*n* = 8). (L) Pantethine and Y27632 suppressed the effect of exercise on improving endothelium‐dependent vasodilation in isolated arteries (*n* = 6). Data are presented as mean ± SEM. *p < 0.05, **p < 0.01.

To investigate whether m/lEV release contributes to the benefits of exercise on vascular endothelial function in vivo, we simulated LSS through exercise and administered Y27632 (5 mg/kg, i.p.) or pantethine (150 mg/kg, i.p.) prior to each exercise to block m/lEV release (Figure 3D, Figure S4D). To reduce contamination by lipoproteins and soluble proteins, plasma samples were purified using a combination of ultracentrifugation and SEC column, which showed that m/lEVs were enriched in fractions 7–9 (Figure 3E,F). NTA analysis of the purified m/lEVs revealed that both pantethine and Y27632 blocked the upregulation of circulating m/lEVs induced by exercise (Figure 3G). The one‐month exercise or Y27632 administration reduced mouse body weight, whereas pantethine had no obvious effect on body weight (Figure S4E). Exercise improved mouse exercise performance, as evidenced by improved motor coordination (Figure S4F), endurance capacity (Figure 3H) and forelimb grip strength (Figure 3I). However, these beneficial effects were diminished by pantethine and Y27632 (Figure 3H,I, Figure S4F). Pantethine and Y27632 also abolished exercise‐induced reductions in aortic intima‐media thickness and blood pressure (Figure 3J,K). Furthermore, pantethine and Y27632 diminished the exercise‐induced improvement in endothelium‐dependent vascular relaxation (Figure 3L). Exercise increased eNOS phosphorylation in the aorta and circulating NO content, which were inhibited by pantethine and Y27632 (Figure S4G,H). In addition, Y27632 and Pantethine abolished the effect of exercise on the reduction of aortic pulse wave velocity (Figure S4I). These results indicate that m/lEV release contributes to exercise‐induced endothelial benefits.

### 3.4 LSS‐induced m/lEV Release Maintains Endothelial Piezo1 Homeostasis

Since Piezo1 translocates to the cell periphery colocalising with the plasma membrane budding sites, it is possible that Piezo1 can be packaged into the m/lEVs. In fact, we observed that Piezo1 was positive in m/lEVs, as evidenced by both immunoelectron and confocal microscopy (Figure 4A,B). To test whether Piezo1 release through m/lEVs is specific to LSS, we utilized A23187 (a calcium ionophore, 500 nM) as a pharmacological inducer of microvesicle shedding. Although A23187 successfully triggered the release of m/lEVs, it failed to promote Piezo1 translocation to the cell periphery (Figure S5A,B). Notably, while LSS or Yoda1 increased the Piezo1 content within isolated m/lEVs, the m/lEVs released in response to A23187 stimulation did not contain Piezo1 (Figure 4C, Figure S5C,D). These results suggest that the packaging of Piezo1 into m/lEVs is a specific response to shear stress. Other types of cells, including HAECs, VSMCs, A549 cells and HepG2 cells, also released Piezo1‐enriched m/lEVs following Yoda1 stimulation (Figure S6). This suggests a potential role of m/lEV release in redistributing Piezo1 and maintenance of intracellular Piezo1 homeostasis.

**FIGURE 4:**
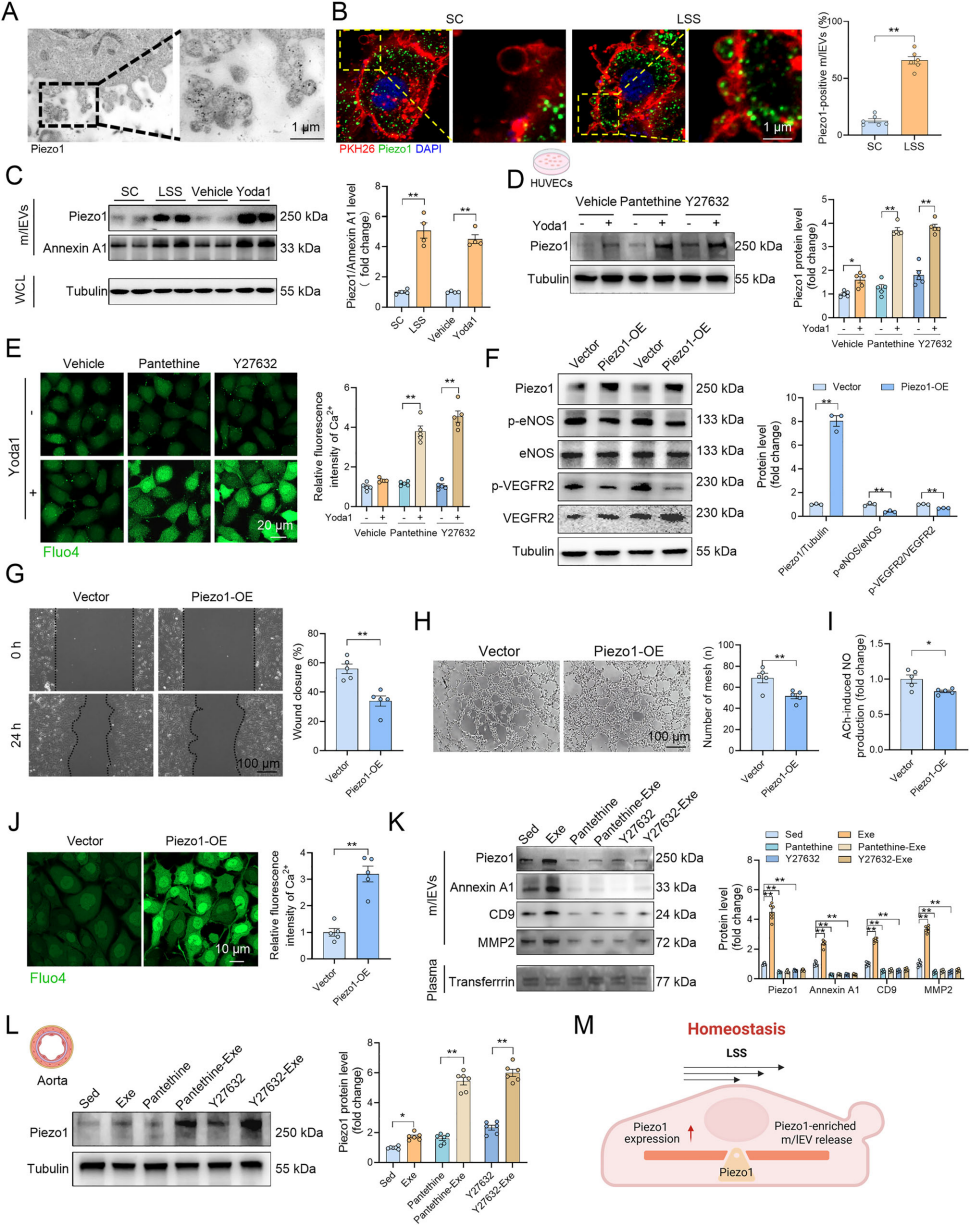
LSS‐induced m/lEV release maintains endothelial Piezo1 homeostasis. (A) Immunoelectron microscope visualized that Piezo1 was packaged into the m/lEVs (*n* = 15 cells from three independent experiments). (B) Left panel: Confocal microscope visualized that LSS (15 dyn/cm^2^ for 300 s) increased the content of Piezo1 in m/lEVs (*n* = 60 cells from six independent experiments). Right panel: LSS increased the percentage of Piezo1‐positive m/lEVs (*n* = 6). (C) Both LSS and Yoda1increased the content of Piezo1 in m/lEVs (*n* = 4). (D) Dual stimulation with Yoda1 (Piezo1 agonist, 2 µM) and m/lEV release inhibitor (500 nM pantethine or 2 µM Y27632) induced Piezo1 over accumulation in HUVECs (*n* = 4). HUVECs were pre‐treated with pantethine or Y27632 for 5 min followed by Yoda1 treatment for 24 h. (E) Dual stimulation with Yoda1 and m/lEV release inhibitor induced Ca^2+^ overload in HUVECs (*n* = 5). (F) Piezo1 overexpression (OE) reduced the phosphorylation of eNOS and VEGFR2 (*n* = 3). Plasmid transfection for Piezo1 OE was performed 60 h prior to the cell functional analysis. (G) Piezo1 OE impaired EC migration (*n* = 5). (H) Piezo1 OE impaired EC tube formation (*n* = 5). (I) Piezo1 OE impaired ACh‐induced NO production (*n* = 5). (J) Piezo1 OE induced Ca^2+^ overload in ECs (*n* = 5). (K) Treatment with either pantethine (150 mg/kg, i.p.) or Y27632 (5 mg/kg, i.p.) abolished the effect of exercise on upregulating Piezo1 in circulating m/lEVs from mice (*n* = 6). The experimental schemes for m/lEV release inhibitor treatment and the exercise model were showed in Figure 3D. (L) Chronic exercise mildly upregulated Piezo1 expression, whereas treatment with either pantethine or Y27632 induced Piezo1 over accumulation in aorta from mice with exercise (*n* = 6). (M) Schematic diagram showing that LSS‐induced m/lEV release contributes to intracellular Piezo1 homeostasis. Data are presented as mean ± SEM. *p < 0.05, **p < 0.01.

We initially tested whether LSS‐induced Piezo1 release plays an important role in maintenance of intracellular Piezo1 homeostasis. Yoda1 mildly increased Piezo1 levels in ECs, consistent with previous studies (Liu et al. 2024) (Figure 4D). However, dual stimulation with Yoda1 and the inhibitor of m/lEV release induced Piezo1 over accumulation in ECs (Figure 4D), accompanied by significant Ca^2+^ overload (Figure 4E). Consistently, Piezo1 overexpression revealed that excessive Piezo1 induced endothelial dysfunction. Piezo1 overexpression reduced the phosphorylation of eNOS and VEGFR2 (Figure 4F), impaired endothelial cell migration (Figure 4G) and tube formation (Figure 4H), and diminished NO production (Figure 4I). Furthermore, Piezo1 overexpression directly caused endothelial Ca^2+^ overload (Figure 4J). We next determined whether m/lEV inhibition induces pathological Piezo1 accumulation in vivo. Pantethine and Y27632 treatment inhibited the effect of exercise on upregulation of Piezo1 in circulating m/lEVs (Figure 4K). In isolated aorta from mice, the combination of exercise and treatment with either pantethine or Y27632 provoked Piezo1 over accumulation (Figure 4L). These results demonstrate that LSS‐induced m/lEV release contributes to maintain intracellular Piezo1 homeostasis in ECs (Figure 4M).

### 3.5 LSS Induces Piezo1 Trafficking From ECs to Splenic Capsule via m/lEVs

To investigate the origin of exercise‐derived m/lEVs, we used mice with endothelial‐specific (Piezo1_EC_^−/−^), SMC‐specific (Piezo1_SMC_^−/−^) and hepatocyte‐specific (Piezo1_Hep_^−/−^) deletion of Piezo1 (Figure 5A, Figure S7A,B). Exercise increased circulating m/lEVs and Piezo1 content in isolated m/lEVs, and Piezo1_EC_^−/−^ abolished the effect of exercise on upregulation of circulating m/lEVs and Piezo1 content in isolated m/lEVs (Figure 5B). Whereas Piezo1_SMC_^−/–^ and Piezo1_Hep_^−/−^ did not affect the effect of exercise on upregulation of circulating m/lEVs (Figure 5B, Figure S7C). Furthermore, we observed an increase in CD31 in exercise‐induced m/lEVs, but not asialoglycoprotein receptor (ASGPR, a hepatocyte marker) (Figure S7D). These results suggest that the vascular ECs are a primary source of exercise‐induced circulating m/lEVs.

**FIGURE 5:**
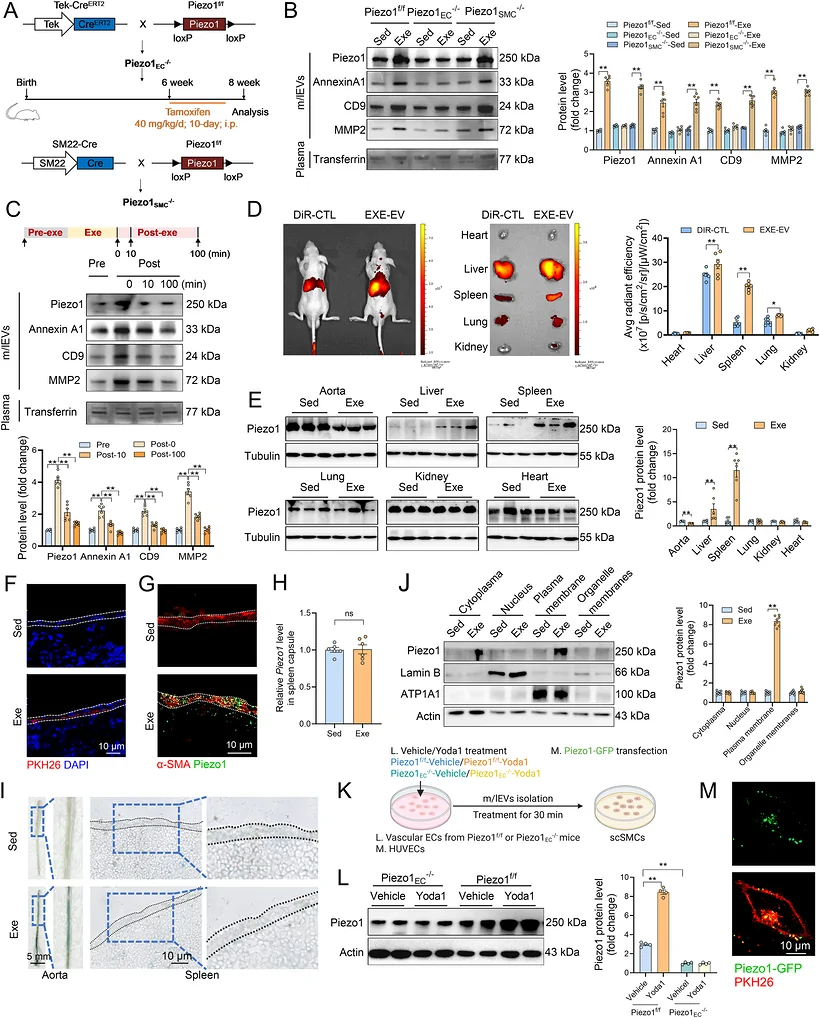
Exercise induces Piezo1 translocation from vascular endothelial cells to the splenic capsule. (A) Schematic of genetically engineered mouse model. (B) Acute exercise‐induced upregulation of Piezo1, Annexin A1, CD9 and MMP2 in circulating m/lEVs was blocked in Piezo1_EC_^−/−^ mice (*n* = 6). (C) Upper panel: The protocol for collecting m/lEVs at baseline, immediately post‐exercise and at 10 and 100 min of recovery. Lower panel: Exercise induced a significant increase in circulating m/lEV levels, which declined post‐exercise (*n* = 6). (D) In vivo imaging identified the spleen as a primary target of exercise‐derived m/lEVs (*n* = 6). DiR, a lipophilic dye for labelling m/lEVs to monitor their in vivo distribution. An equivalent concentration of free DiR dye in PBS was prepared as control. (E) Acute exercise decreased Piezo1 content in the aorta and increased Piezo1 content in spleen (*n* = 6). (F) Acute exercise‐derived and PKH26‐labeled m/lEVs were enriched in the splenic capsule but not splenic parenchyma (*n* = 6). PKH26, a lipophilic membrane dye for labelling m/lEVs isolated from the plasma of exercised mice. Splenic capsule is outlined by white dashed lines. (G) Piezo1 were enriched in the splenic capsule 30 min post‐exercise (*n* = 6). α‐SMA, a marker for smooth muscle cells, labelling the splenic capsule. (H) qPCR analysis showed no significant change of *Piezo1* mRNA expression in the splenic capsule following acute exercise (*n* = 6). (I) In LacZ Piezo1 reporter mice (Piezo1^LacZ/+^), β‐galactosidase staining revealed undetectable endogenous *Piezo1* expression in the spleen in mice with and without acute exercise, whereas it was increased in the aorta following exercise. Splenic capsule is outlined by dashed lines. (*n* = 6). (J) Piezo1 was located across various subcellular fractions of the splenic capsule and enriched in the plasma membrane fraction following exercise (*n* = 8). Lamin B, ATPase Na^+^/K^+^ transporting subunit alpha 1 (ATP1A1), and actin were used as markers for the nucleus, plasma membrane and cytoplasm, respectively. (K) Vascular ECs were isolated from Piezo1^f/f^ and Piezo1_EC_^−/−^ mice and treated with Yoda1 for 24 h before m/lEV collection. HUVECs were transfected with Piezo1‐GFP plasmid for 60 h before m/lEV collection. m/lEVs were isolated from vascular ECs or HUVECs and incubated with scSMCs for 30 min. (L) Piezo1 protein levels were increased in recipient scSMCs following incubation with m/lEVs from Yoda1‐stimulated wildtype ECs but not Piezo1 knockout ECs (*n* = 4). (M) m/lEV‐carried Piezo1 localized to the plasma membrane and the intracellular space of scSMCs (*n* = 4). Data are presented as mean ± SEM. **p < 0.01.

Mice were subjected to a single bout of exercise (18 m/min for 60 min) and circulating m/lEVs were isolated post‐exercise at different time points (Figure 5C). Acute exercise elevated circulating m/lEV, which were progressively declined post exercise and returned to basal level at 100 min post‐exercise (Figure 5C), indicating an endogenous uptake of m/lEVs by other cells. To trace exercise‐induced m/lEVs, isolated m/lEVs from a mouse with acute exercise were labelled with DiR (5 µM) and injected (resuspended in 200 µL PBS, i.v.) into the other mouse without exercise (Figure 5D). An equivalent concentration of free DiR dye in PBS (DiR‐CTL) was prepared as control. In vivo small animal imaging revealed preferential uptake of exercise‐derived m/lEVs by the spleen, liver and lungs. The spleen emerged as a primary target organ for exercise‐derived m/lEVs (Figure 5D). Western blot analyses demonstrated low basal Piezo1 protein levels in the spleen and liver, with spleen as an interesting organ which Piezo1 was upregulated more obviously post‐exercise (Figure 5E). Whereas exercise did not increase the mRNA level of *Piezo1* in the liver and the spleen (Figure S7E). In addition, a single bout of exercise concurrently reduced aortic Piezo1 levels (Figure 5E). Notably, these in vitro‐labelled and exercise‐derived m/lEVs and Piezo1 were enriched in the splenic capsule but not splenic parenchyma (Figure 5F,G). qRT‐PCR and Piezo1^lacZ/+^ mice demonstrated that *Piezo1* was poorly expressed in the splenic capsule and its expression could not be evaluated by exercise (Figure 5H,I), whereas aortic Piezo1 expression was significantly upregulated after exercise (Figure 5I). These findings demonstrate a selective homing of exercise‐derived m/lEVs to the splenic capsule inside the spleen.

Spleen was previous identified as one of the major organs responsible for systemic EV clearance, primarily through capture and degradation by macrophages in the red pulp (Kang et al. 2021). However, we observed the enrichment of EVs in the splenic capsule, a thin outer boundary of the spleen. The splenic capsule is structurally supported by a specialized SMC layer, which mediates spleen contraction. To determine the subcellular distribution of Piezo1 in splenic capsule cells, we lysed splenic capsules and performed subcellular fractionation experiments. The result demonstrated that Piezo1 was located across various subcellular fractions of the splenic capsule, and enriched in the plasma membrane fraction following exercise (Figure 5J). To validate this phenomenon in vitro, we carried out m/lEV uptake and localisation assays in isolated scSMCs (Figure 5K). Incubation with m/lEVs derived from Yoda1‐stimulated wildtype ECs increased Piezo1 protein levels in recipient scSMCs, whereas this effect was abolished when using m/lEVs from ECs from Piezo1_EC_^−/−^ mice (Figure 5L, Figure S7F). To determine m/lEV‐derived Piezo1 localization, HUVECs were transfected with a Piezo1‐GFP plasmid and subjected to LSS stimulation (Figure 5K). m/lEVs isolated from these stimulated cells were incubated with scSMCs (Figure S7G). The m/lEV‐delivered Piezo1 localized at both the plasma membrane and intracellular space of recipient scSMCs (Figure 5M). These results demonstrate that exercise‐derived m/lEVs mediate the delivery of Piezo1 from ECs to the splenic capsule inside the spleen.

### 3.6 Piezo1 Trafficking to Spleen Promotes Spleen Contraction During Exercise

We investigated whether Piezo1 transportation to the splenic capsule regulates spleen contraction by performing ultrasonographic measurements of spleen sizes (Figure 6A). Inhibition of Piezo1 transportation through EC‐specific KO (Piezo1_EC_^−/−^) and pharmacologic inhibition of EV release (Y27632) blocked the exercise‐induced elevation of splenic Piezo1 levels (Figure 6B). Acute exercise induced a 22.90% contraction of the spleen in wild‐type mice, and three strategies to inhibit Piezo1 transport, including EC‐specific KO (Piezo1_EC_^−/−^), Y27632 treatment, and GsMTx4 treatment, decreased the spleen contraction to 12.65%, 9.63% and 9.25%, respectively (Figure 6C). Meanwhile, these three strategies reduced circulating blood volume during exercise (Figure 6D). While short‐duration rotarod testing for motor coordination showed no significant difference (Figure 6E), prolonged endurance rotarod testing demonstrated that inhibiting Piezo1 transportation increased the frequency of falls (Figure 6F). Furthermore, treadmill endurance assessments confirmed that suppression of Piezo1 transportation impaired exercise endurance (Figure 6G). Additionally, to test whether splenic uptake of m/lEVs is required for spleen contraction, we inhibited endocytosis using Dynasore (a dynamin‐dependent endocytosis inhibitor, 50 µM) to reduce m/lEV uptake in scSMCs (Figure S8A). Dynasore (10 mg/kg, i.p.) reduced the exercise‐induced elevation of Piezo1 on the splenic capsule and decreased the spleen contraction induced by exercise (Figure S8B,C). These findings establish that Piezo1 redistribution to the splenic capsule serves as an important contributor to inducing spleen contraction and maintaining exercise endurance in mice.

**FIGURE 6:**
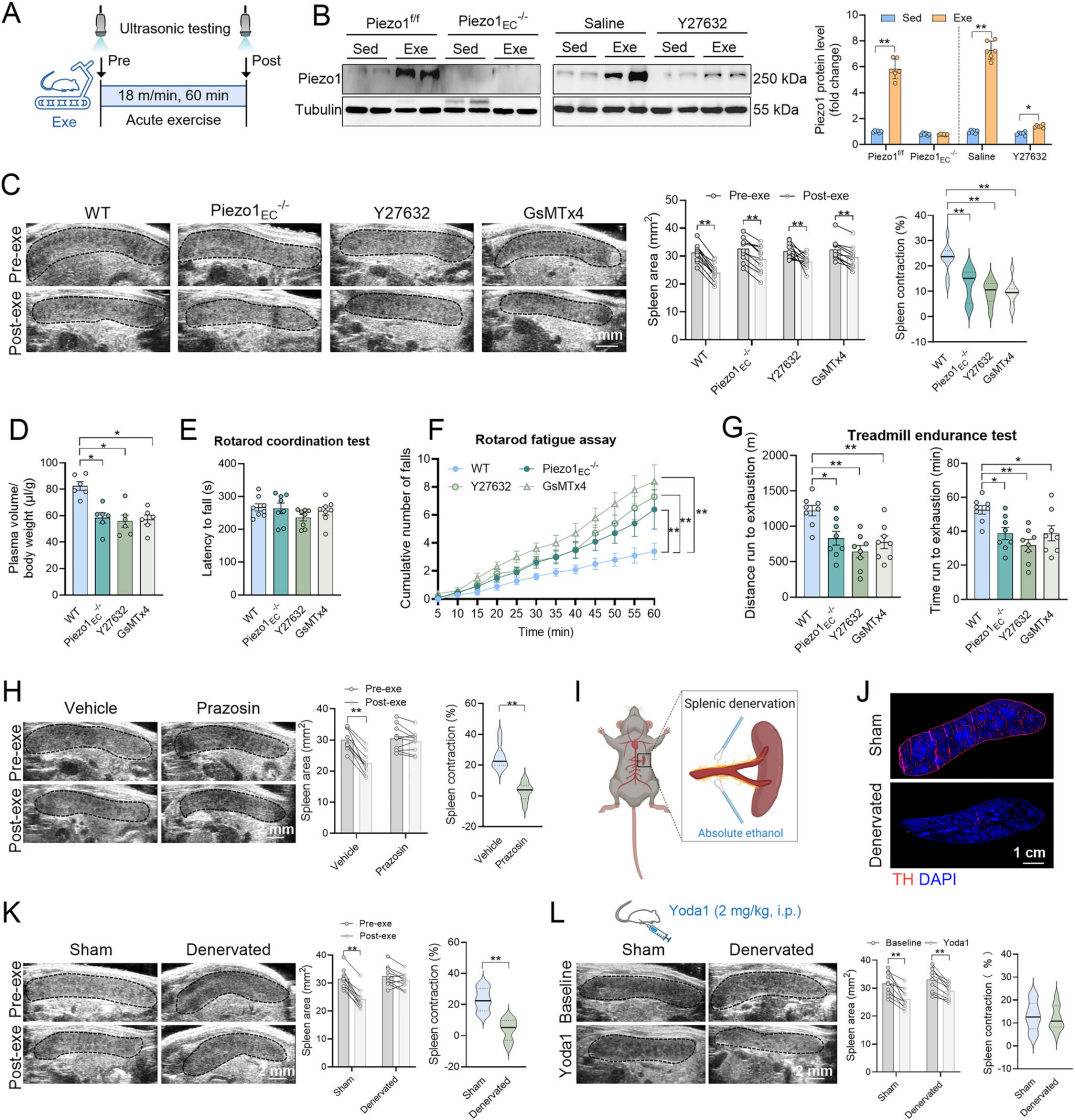
Piezo1 transportation to the splenic capsule promotes spleen contraction and maintains exercise endurance. (A) Schematic diagram of the ultrasonographic measurements before and after acute exercise. (B) Inhibition of Piezo1 transportation through EC‐specific KO (Piezo1_EC_^−/−^) and Y27632 (5 mg/kg, i.p., 30 min prior to exercise) abolished the effect of exercise on upregulating splenic Piezo1 contents (*n* = 6). (C) Piezo1_EC_^−/−^, Y27632 and GsMTx4 (10 mg/kg, i.p., 30 min prior to exercise) suppressed exercise‐induced spleen contraction (*n* = 10). Left panel: Representative ultrasound images of the spleen pre‐ and post‐exercise. Middle panel: Quantification of spleen area pre‐ and post‐exercise from ultrasound images. Right panel: Percentage change in spleen contraction in response to exercise. (D) Piezo1_EC_^−/−^, Y27632 and GsMTx4 decreased circulating blood volume during exercise (*n* = 6). (E) Piezo1_EC_^−/−^, Y27632 and GsMTx4 showed no significant effect on motor coordination quantified from average latency to fall from rotarod (*n* = 8). (F, G) Piezo1_EC_^−/−^, Y27632 and GsMTx4 decreased exercise endurance in mice, as detected by cumulative numbers of falls binned every 5 min of mice during 1 h rotarod endurance test (F), and distance and time run to exhaustion during a treadmill endurance test (G) (*n* = 8). (H) α1AR antagonist (Prazosin, 1 mg/kg, i.p., 30 min prior to exercise) abolished exercise‐induced spleen contraction (*n* = 10). (I) Schematic illustration of splenic denervation via absolute ethanol ablation. (J) Representative images of sham‐operated (upper) and denervated (lower) spleens 5 weeks post‐surgery. Tyrosine hydroxylase (TH) was used to label neural fibers. (K) Splenic denervation abolished exercise‐induced spleen contraction (*n* = 10). (L) Yoda1 (2 mg/kg, i.p.) induced spleen contraction even with ablation of the splenic nerve plexus (*n* = 10). Ultrasonographic measurements were performed before and at 1 h after Yoda1 injection. Data are presented as mean or mean ± SEM. *p < 0.05, **p < 0.01.

The splenic nerve comprises 98% sympathetic fibres, with contraction predominantly initiated by sympathetic activation via norepinephrine (NE) release (Felten et al. 1985). Inhibition of Piezo1 transport also inhibited NE‐induced (adrenergic receptor [AR] agonist, 1 mg/kg, i.p.) spleen contraction (Figure S8D). α1‐AR constitutes the critical signalling molecule for sympathetic‐induced spleen contraction. To investigate the relationship between Piezo1 and α1AR in spleen contraction, we employed Prazosin (α1AR antagonist, 1 mg/kg, i.p.), which totally abolished exercise‐induced spleen contraction (Figure 6H). Furthermore, surgical ablation of the splenic nerve plexus using absolute ethanol (Figure 6I), as validated by tyrosine hydroxylase staining (Figure 6J), completely abolished exercise‐induced contraction (Figure 6K). Chemical denervation by 6‐hydroxydopamine hydrochloride (6‐OHDA, 100 mg/kg, i.p.) also completely abolished exercise‐induced contraction (Figure S8E). However, Yoda1 alone induced spleen contraction even with ablation of the splenic nerve plexus or chemical denervation by 6‐OHDA (Figure 6L, Figure S8F). These results suggest that splenic capsule‐localized Piezo1 functions as a downstream regulator of sympathetic α1AR activation to enhance spleen contraction.

### 3.7 Piezo1 Acts as a Downstream Molecule of α1AR Through Protein Kinase C (PKC) Signalling

We further investigated the mechanism underlying α1AR and Piezo1 in vitro. m/lEVs released in response to LSS stimulation were isolated and cultured with scSMCs (Figure 7A). NE (AR agonist, 100 nM)‐induced scSMC contraction as detected by MLC phosphorylation was totally abolished by prazosin (α1AR antagonist, 10 µM), but only partially inhibited by GsMTx4 (Piezo1 inhibitor, 5 µM). Conversely, Yoda1 (Piezo1 agonist, 2 µM)‐induced scSMC contraction as detected by MLC phosphorylation was totally blocked by GsMTx4 but remained unaffected by prazosin (Figure 7B). These findings were further corroborated in collagen contraction assays (Figure 7C). These in vitro results reinforce the notion that Piezo1 acts as a downstream molecule of α1AR and partially mediates NE‐induced scSMC contraction.

**FIGURE 7:**
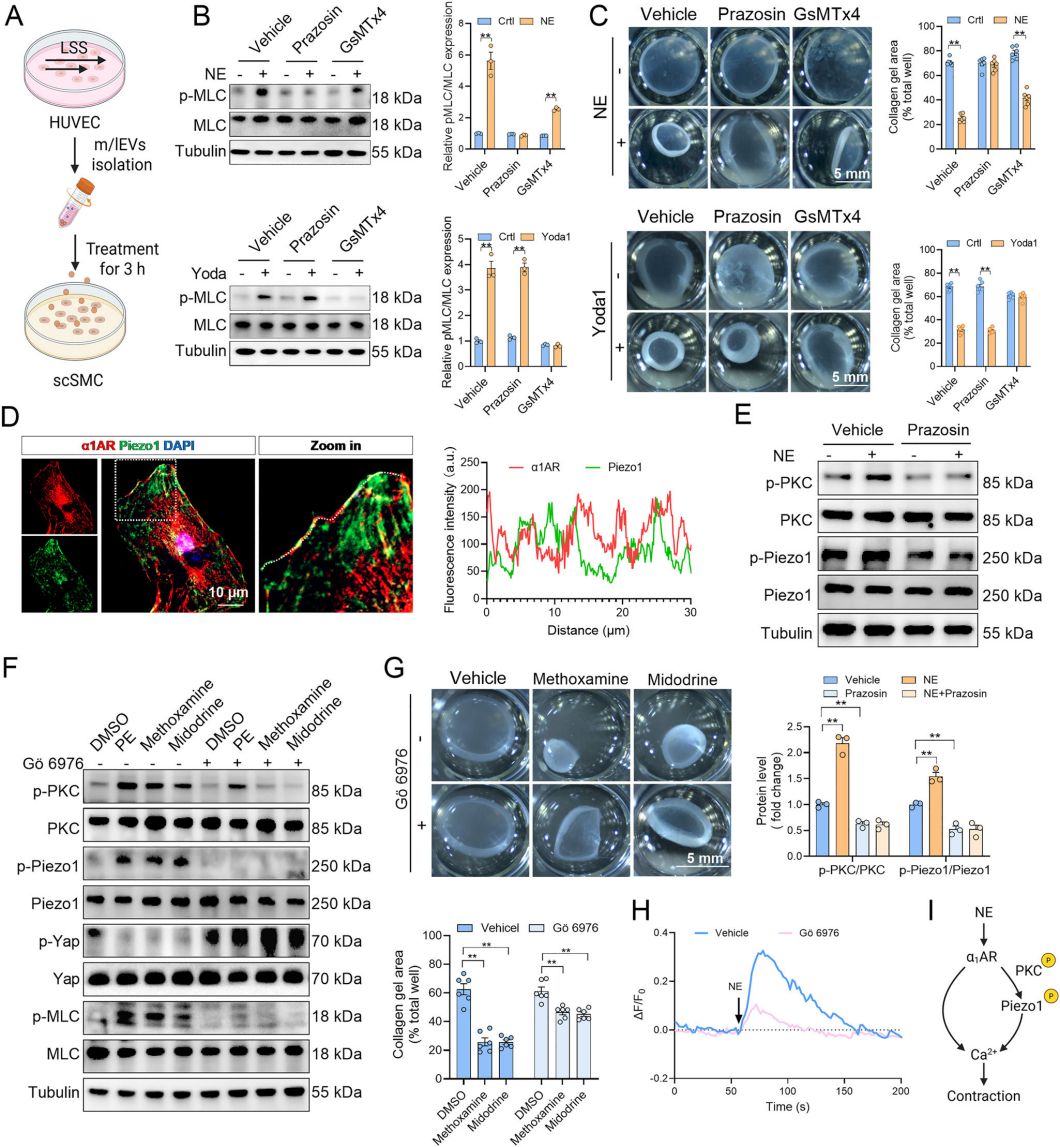
Piezo1 acts as a downstream molecule of α1 adrenergic receptor (α1AR) through PKC signalling. (A) Isolated scSMCs were co‐cultured with m/lEVs derived from HUVECs. HUVECs were stimulated with LSS at 15 dyn/cm^2^ for 24 h prior to m/lEV isolation. (B) Upper panel: MLC phosphorylation induced by norepinephrine (NE, α1AR agonist, 100 nM) was completely abolished by prazosin (α1AR antagonist, 10 µM) but only partially inhibited by GsMTx4 (Piezo1 inhibitor, 5 µM). Lower panel: MLC phosphorylation induced by Yoda1 (Piezo1 agonist, 2 µM) was fully abolished by GsMTx4 but remained unaffected by prazosin (*n* = 3). (C) Upper panel: NE‐induced collagen contraction was abolished by prazosin but only partially inhibited by GsMTx4. Lower panel: Yoda1‐induced collagen contraction was fully blocked by GsMTx4 but remained unaffected by prazosin (*n* = 6). (D) Confocal imaging revealed no co‐localisation between α1AR and Piezo1 (*n* = 5). (E) Prazosin inhibited NE‐induced phosphorylation of PKC and Piezo1 (*n* = 3). (F) Gö 6976 (PKC inhibitor, 1 µM) blocked the effects of the α1AR agonists (phenylephrine [PE], 100 nM. methoxamine, 1 µM. midodrine, 30 µM), including the phosphorylation of PKC, Piezo, MLC and the dephosphorylation of YAP. (G) Gö 6976 reduced collagen contraction induced by methoxamine and midodrine (*n* = 6). (H) Gö 6976 suppressed NE‐induced Ca^2+^calcium influx (*n* = 5). (I) Schematic diagram showing that α1AR and Piezo1 cooperatively regulate scSMC contraction, where Piezo1 in scMSCs acts as a downstream regulator of α1AR activation. All inhibitors were added 5 min prior to agonist treatment. For Western blot analysis, HUVECs were treated with a specific agonist for 30 min. For collagen gel contraction assay, HUVECs were treated with specific agonist for 24 h. Data are presented as mean ± SEM. **p < 0.01.

Although α1AR and Piezo1 primarily function at the plasma membrane, confocal imaging revealed no co‐localisation between α1AR and Piezo1 (Figure 7D). Prior studies indicate that PKC activates Piezo1 via phosphorylation at serine‐1612 (S1612) (Zhang et al. 2024) and PKC is a core effector kinase downstream of α1AR, which activates phospholipase C via Gq protein, generating diacylglycerol that directly activates PKC to promote SMCs contraction. We demonstrated that NE promoted phosphorylation of both PKC and Piezo1, an effect suppressed by the α1AR inhibitor prazosin (Figure 7E). Additionally, three distinct α1AR agonists (phenylephrine, 100 nM; methoxamine, 1 µM; midodrine, 30 µM) were used to validate the functional relationship between α1AR, PKC and Piezo1. All three α1AR agonists induced phosphorylation of PKC, Piezo1 and MLC, while reducing phosphorylation of Yap (the downstream molecule of Piezo1 activation), and these effects were blocked by Gö 6976 (PKC inhibitor, 1 µM) (Figure 7F). Additionally, collagen contraction assays confirmed that the α1AR agonists methoxamine and midodrine promoted scSMC contraction, which were blocked by Gö 6976 (Figure 7G). Furthermore, NE‐induced Ca^2+^ influx was partially inhibited by Gö 6976 (Figure 7H). These results establish that α1AR and Piezo1 cooperatively regulate scSMC contraction, where Piezo1 acts as a downstream regulator of α1AR activation (Figure 7I).

### 3.8 Piezo1 Transportation and Spleen Contraction During Exercise in Healthy Adults

Next, we recruited 20 healthy volunteers to test the Piezo1 transportation and spleen contraction during exercise (Figure 8A) (Table S1). All adults were subjected to a single dose of exercise (Figure 8A). Since all current clinical methods for calculating spleen volume assumed the spleen to be an ellipsoid (Ogunleye et al. 2023), an method we considered inaccurate given the spleen's non‐ellipsoidal morphology, we instead measured spleen size using ultrasound to assess its area in both the coronal and transverse planes. Our findings revealed that acute exercise significantly reduced the spleen area in both coronal and transverse planes by 26.83% and 22.86%, respectively, and markedly increased heart rate from 68.15 to 173.60 bpm, cardiac output from 3.57 to 5.67 L/min, and popliteal artery blood flow from 11.96 to 23.19 mL/s (Figure 8B, Figure S9A,B). Immunoelectron microscopy confirmed the presence of Piezo1 in these m/lEVs (Figure 8C). NTA revealed that the concentration of circulating m/lEVs was increased after exercise (Figure 8D). Western blot analysis of m/lEVs showed substantial increases in Piezo1 post‐exercise, as well as the microvesicle markers Annexin A1 and CD9 (Figure 8E). Acute exercise increased the levels of CD9 and Piezo1 in m/lEVs by 1.61‐ and 1.65‐fold, respectively, as measured by ELISA kits (Figure 8F,G). Correlation analysis demonstrated that spleen contraction in the coronal plane correlated with exercise‐induced fold changes in CD9, Piezo1, heart rate, maximal heart rate and popliteal artery blood flow (Figure 8H). Similarly, transverse plane spleen contraction correlated with fold changes in CD9, Piezo1, heart rate and popliteal artery blood flow following exercise (Figure 8H). To control multicollinearity and identify core predictors, the study introduced regularized regression following correlation analysis. Under the Ridge model, CD9 (Post/Pre) demonstrated the highest importance in the spleen contraction of coronal panel, while Piezo1 (Post/Pre) showed higher importance in the spleen contraction of transverse panel (Figure 8I,J). Similarly, in the Lasso model, CD9 (Post/Pre) remained the most significant predictor in the spleen contraction in the coronal plane, whereas Piezo1 (Post/Pre) exhibited greater importance in the spleen contraction of transverse panel (Figure S9C,D). These results demonstrate a correlation between circulating Piezo1 levels and the degree of spleen contraction in human volunteers.

**FIGURE 8:**
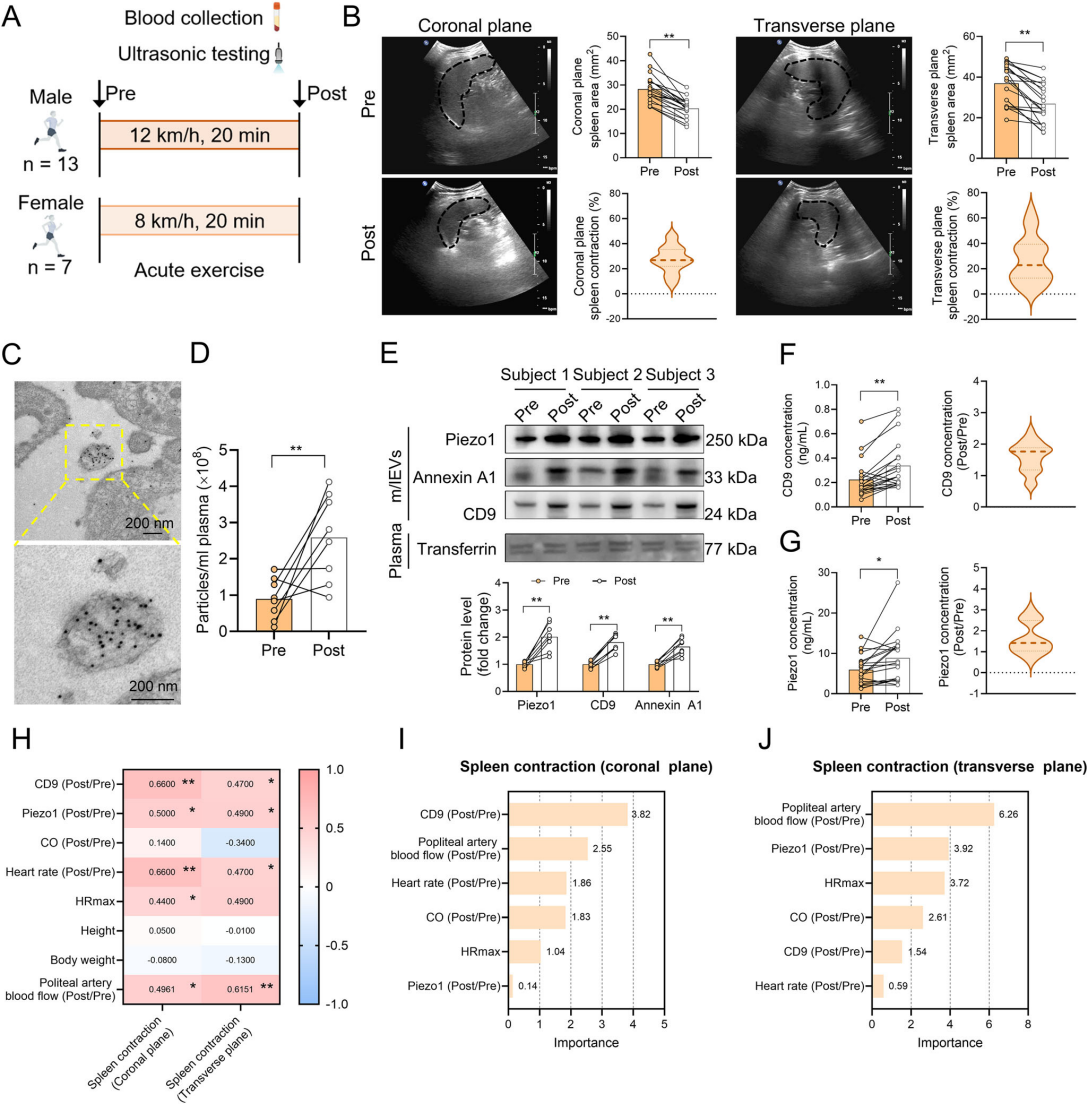
Elevated Piezo1 levels in m/lEVs are associated with spleen contraction during exercise in healthy adults. (A) Schematic of the experimental protocol for a single bout of treadmill running in healthy adults. (B) Acute exercise reduced the spleen area in both coronal and transverse planes (*n* = 20). (C) Piezo1 was visualized by immunoelectron microscope in m/lEVs isolated from blood. (D) The number of circulating m/lEVs was increased after exercise. (E) Exercise upregulated Piezo1 and microvesicle biomarkers (CD9 and Annexin A1) in m/lEVs as detected by western blot (*n* = 6). (F) Exercise increased CD9 level in circulating m/lEVs as detected by ELISA kits (*n* = 20). (G) Exercise upregulated Piezo1 level in circulating m/lEV as measurement by ELISA kits (*n* = 20). (H) Correlation heatmap of spleen contraction (coronal/transverse planes) and candidate biomarkers (CD9, Piezo1, dorsal pedal artery blood flow, etc.). Color intensity denotes correlation strength (red: positive, blue: negative). Values represent Pearson's correlation coefficients (*r*), with asterisks indicating statistical significance (*n* = 20). (I) Feature importance ranking in the Ridge model for predicting coronal plane spleen contraction magnitude. Bar length represents contribution weight (*n* = 20). (J) Feature importance ranking for transverse plane spleen contraction prediction (*n* = 20). Data are presented as mean in the bar graph. Data are presented as the median with interquartile range in the violin plots. *p < 0.05, **p < 0.01.

## 4 Discussion

Vascular ECs lining the blood vessel walls are constantly exposed to haemodynamic shear stress. This mechanical force modulates endothelial homeostasis by regulating a range of cellular processes, including proliferation, apoptosis and inflammatory responses (Tamargo et al. 2023). Previous studies have established that shear stress serves as a potent trigger for the release of EVs (Thompson and Papoutsakis 2023). Different from previous studies that focus on small EVs, we found that shear stress induces m/lEV release from ECs in a dose‐dependent manner, and ECs are the major source of m/lEV release induced by exercise. These m/lEVs display microvesicle‐like features, including their formation via plasma membrane budding, larger size and microvesicle biomarker positivity. Microvesicle biogenesis requires several molecular rearrangements within the plasma membrane, including Ca^2+^‐induced changes in lipid components and protein composition. Ca^2+^‐dependent enzymatic machineries include aminophospholipid translocases (flippases and floppases), scramblases, CaMKII and calpain (van Niel et al. 2018; Dixson et al. 2023). Here, we demonstrated that Piezo1 activation mediates shear stress‐induced m/lEV release, which activates CAMKII, leading to MLC phosphorylation and the subsequent shedding of microvesicles from the plasma membrane.

Mechanosensitive receptors localized on the plasma membrane of ECs are responsible for converting shear stress‐derived biomechanical stimuli into intracellular biochemical signals. These mechanosensitive receptors comprise a diverse array of receptors and proteins, such as GPCRs, NOTCH1, P2X purinoreceptor 4 (P2X4), Piezo1 and plexin D1 (He et al. 2022). Upon activation, they initiate multiple signalling pathways to regulate various endothelial functions (Cheng et al. 2025). Among the multitude of mechanosensitive receptors, the Piezo1 channel has emerged as a critical sensor for shear stress. Here, we found that Piezo1 is not only a signal in initiating m/lEV release; it is also involved in other processes during EV release, including translocation of Piezo1 to the cell periphery and release of Piezo1 in m/lEVs. These two processes are regulated by the same signalling pathway, namely Piezo1‐Ca^2+^‐CaMKII signalling. The difference is that Piezo1 translocation does not depend on MLC phosphorylation, whereas m/lEV release does. Piezo1 translocation to the cell periphery facilitates its packaging into budding microvesicles and drives the release of Piezo1‐enriched m/lEVs. Consistent with previous studies, exercise not only alters EV quantity, but also modifies their molecular cargo (Hou et al. 2019; Lou et al. 2022). Recent omics analyses indicate that exercise‐induced changes in EV content are involved in signal transduction, immune regulation, metabolic processes and oxidative stress (Chong et al. 2024; Giloteaux et al. 2024; Whitham et al. 2018). Our previous findings have similarly demonstrated that circulating small EVs derived from aerobic exercise confer profound and sustained cardioprotection against myocardial ischemia/reperfusion injury (Hou et al. 2019). This study extends these advances by showing that Piezo1 activation‐induced m/lEVs shuttle Piezo1 protein, acting as a negative‐feedback mechanism in mechanical signalling transduction.

Haemodynamic shear stress is supposed to be an initial signal which mediates exercise‐afforded benefits in both ECs and other cells (Green et al. 2017; Cheng et al. 2025). Acute exercise increases the level of circulating EVs, where ECs are one of the major sources (Brahmer et al. 2019; Ma et al. 2018). Here, we found that ECs are the major source of circulating m/lEVs and Piezo1 protein in response to acute exercise. Only EC‐specific knockout of Piezo1 (compared to hepatocyte‐specific knockout and SMC‐specific knockout) abolished the effect of exercise on upregulation of circulating Piezo1‐riched m/lEV. More importantly, we identified shear stress‐induced m/lEV release as a novel mechanism that fine‐tunes endothelial Piezo1 homeostasis and endothelial function. Specifically, exercise not only upregulates Piezo1 expression but also promotes the Piezo1 release through m/lEVs, thereby maintaining physiological intracellular Piezo1 levels. Inhibition of m/lEV release in response to shear stress or exercise through pharmacological strategies diminished exercise‐induced endothelial and overall benefits. It should be noted that pantethine and Y27632 do not merely inhibit m/lEV release, as they target upstream signals governing m/lEV secretion. This homeostatic process may underlie the paradox in the previous studies: while some studies demonstrate that Piezo1 overexpression enhances endothelial function, others indicate it disrupts cellular homeostasis (Bartoli et al. 2022; Beech 2025; Zhang, Leng, et al. 2024). These results suggest that Piezo1 exerts dichotomous, threshold‐dependent regulation of endothelial function: suprathreshold accumulation impairs endothelial function, whereas subthreshold expression enhances it, and highlight the role of m/lEV release as a physiological process in maintenance of intracellular Piezo1 homeostasis.

Exercise‐induced haemodynamic changes facilitate intercellular communication between ECs and other cells, contributing to the systemic benefits of exercise (Llorente et al. 2024). Here, we identified the splenic capsule as the primary target for exercise‐induced m/lEVs. The splenic capsule contains smooth muscle cells primarily involved in regulating spleen size and releasing stored blood from the spleen (Stewart and McKenzie 2002). At rest, the spleen remains relaxed and completely isolated from circulating blood, capable of storing approximately 5% of the body's total blood volume. The haematocrit level in the spleen is relatively high, with red blood cell counts reaching about 20% of the body's total erythrocyte reserves (Mostert et al. 1968; Fudenberg et al. 1961). Functioning as the body's ‛emergency blood reservoir’, the spleen releases high‐concentration red blood cell reserve during physiological stress (such as exercise), rapidly increasing circulating blood volume and oxygen delivery capacity (Stewart and McKenzie 2002). In this study, we investigated whether Piezo1 cooperates with α1AR to promote spleen contraction. Previous research has shown that Piezo1 can be activated independently of mechanical forces via phosphorylation at serine 1612 (Zhang, Bi, et al. 2024). Our study demonstrated that Piezo1 serves as a downstream signalling molecule of α1AR. Specifically, we showed that α1AR activation promotes the phosphorylation of spleen‐localized Piezo1 via PKC, thereby activating Piezo1 and leading to subsequent smooth muscle contraction through calcium signalling. Importantly, we showed that this process is involved in the regulation of exercise endurance. Inhibition of exercise‐induced m/lEV release from ECs or inhibition of m/lEV uptake impairs spleen contraction and exercise endurance. Furthermore, positive correlations between exercise‐induced increases in circulating Piezo1‐positive m/lEVs and the degree of spleen contraction were also observed in human volunteers.

Taken together, our study demonstrates that exercise‐induced shear stress triggers the release of Piezo1‐enriched m/lEVs from ECs via Piezo1‐Ca^2+^‐CaMKII‐MLC signalling. This process not only contributes to maintaining endothelial function but also facilitates spleen contraction during exercise. Mechanistically, Piezo1 release maintains intracellular Piezo1 homeostasis in ECs, and Piezo1 translocation to the splenic capsule acts as a downstream regulator of α1AR signalling to enhance spleen contraction. These findings unveil a vasculo‐splenic axis mediated by EC‐derived m/lEVs during exercise, which shuttle Piezo1 to maintain endothelial function and exercise endurance, highlighting a critical role of Piezo1 in inter‐organ communication.

## Author Contributions

**Changyang Xing**: methodology. **Lijun Yuan**: methodology. **Zihang Feng**: investigation. **Yang Hu**: formal analysis, investigation. **Shengpeng Wang**: methodology. **Jing Lou**: conceptualization, funding acquisition, investigation, writing – original draft, formal analysis, methodology, data curation. **Jia Li**: supervision. **Han Li**: investigation, data curation. **Mingzong Yang**: investigation, formal analysis. **Xuyun Liu**: investigation, formal analysis. **Feng Gao**: conceptualization, funding acquisition, project administration, supervision. **Yuxin Xi**: investigation, methodology. **Xing Zhang**: conceptualization, funding acquisition, project administration, writing – review and editing, supervision.

## Conflicts of Interest

The authors declare no conflicts of interest.

## Supporting information



Supporting information: jev270375‐sup‐0001‐SuppMat.docx

Supporting information: jev270375‐sup‐0002‐VideoS1.mp4

## Data Availability

The data that support the findings of this study are available from the corresponding author upon reasonable request.
