# Supplementary material for: Shear Stress‐Stimulated Trafficking of Endothelial Piezo1 to Splenic Capsule Boosts Spleen Contraction and Exercise Endurance
Source: J Extracell Vesicles. 2026 Sep 28;15(10):e70375. doi: 10.1002/jev2.70375 (PMC13619147; doi:10.1002/jev2.70375)
Supplement: Supplementary file 1 [file JEV2-15-e70375-s001.docx]

**Supplementary materials**

**Supplementary methods**

**m/lEVs distribution analysis *in vivo***

The isolated m/lEVs from one mouse were suspended in 200 μL phosphate buffered saline (PBS) and labeled with DiR (HY-D1048, MCE, USA) at a final concentration of 5 μM for 30 min at 37°C. As a negative control, an equivalent concentration of free DiR dye in PBS (DiR-CTL) was prepared under identical conditions. DiR-CTL or labeled m/lEVs were intravenously administered into recipient mice via the tail vein (200 μL/mouse). Mice were euthanized 30 min post-injection, and major organs (heart, liver, spleen, lungs, kidneys) were harvested for *ex vivo* imaging. Fluorescence distribution was quantified using an in vivo imaging system (IVIS) Spectrum imaging (PerkinElmer, USA).

**Circulating blood volume measurement**

The circulating blood volume was determined using the Evans Blue dye dilution method. Briefly, a 2% (w/v) solution of Evans Blue (Sigma-Aldrich, USA) was prepared in saline. A standard curve was established by measuring the absorbance of known concentrations of this EB solution at 620 nm using a microplate reader. Immediately upon completion of the exercise protocol, the 2% Evans Blue solution was administered via the retro-orbital plexus. Blood samples were subsequently collected from the tail vein at 20-, 40-, 60-, 80-, and 100-min post-injection. Each blood sample was centrifuged, and the absorbance of the plasma supernatant was measured at 620 nm. The concentration of Evans Blue in each plasma sample was calculated from the standard curve. The dilution factor of Evans Blue at time zero (the end of exercise) was determined by extrapolating the clearance curve of Evans Blue concentration over time, which was used to calculate the circulating blood volume at the point of exercise cessation.

**Culture of other types of cells**

Vascular smooth muscle cells (VSMCs) were isolated from the mice aorta. Aortic explants were aseptically prepared and minced into small fragments (approximately 1 mm³). These tissue fragments were evenly distributed onto the surface of a culture flask. A minimal volume of Dulbecco's Modified Eagle Medium (HDMEM; Sigma-Aldrich, USA) with 10% fetal bovine serum (FBS; Gibco, USA) and 1% penicillin/streptomycin solution (Corning, USA) was carefully added to just cover the explants, and the flask was left undisturbed in a 37°C, 5% CO₂ incubator for several hours to allow for firm attachment. Subsequently, additional medium was gently added. VSMCs were observed to migrate from the explants after 3-5 days. The explants were removed once a sufficient outgrowth of cells was achieved, and the cells were subsequently passaged upon reaching confluence. Primary endothelial cells (ECs) were isolated from mice thoracic aortas. Briefly, thoracic aortas were excised and opened longitudinally and digested with 2 mg/mL collagenase type II at 37 ℃ for 45 min. ECs were gently detached from the luminal surface, collected by centrifugation, and purified by magnetic-activated cell sorting using anti-platelet endothelial cell adhesion molecule (CD31) MicroBeads (130-097-418, Miltenyi Biotec, Germany). Purified cells were seeded onto fibronectin-coated dishes (F1141, Sigma-Aldrich, USA) and cultured in EC growth medium (1001, ScienCell, USA). The purity of ECs was confirmed through immunofluorescence staining for CD31 (1:200, ab182981, Abcam, UK) (>70%). Additionally, A549 cells, HepG2 cells, and human aortic endothelial cells (HAECs), sourced from the American Type Culture Collection, were also maintained in the same HDMEM. Routine PCR-based mycoplasma screening was performed every two weeks on all cell cultures used in this study, and no positive results were obtained throughout the experimental period.

**Measurement of nitric oxide**

For the assessment of ACh-induced nitric oxide (NO) production, nitrate and nitrite concentrations were quantified in both plasma and cell supernatants using a fluorometric nitrate/nitrite assay kit (780051, Cayman Chemical) according to the manufacturer’s protocol. For plasma nitrate analysis, blood samples obtained via venous puncture were immediately centrifuged at 6,800 × g for 5 minutes at 4°C, and 20 μL of the resulting supernatant was used for nitrate quantification. For cell supernatant analysis, cells were pretreated with acetylcholine (Ach, 10 μM) for 30 minutes at 37°C. After treatment, 40 μL of the supernatant was collected for nitrate/nitrite measurement.

**H&E staining**

Tissue specimens were fixed in 4% paraformaldehyde, paraffin-embedded, and sectioned at 4 μm. After deparaffinization and rehydration, sections were stained with hematoxylin (5 min) and eosin (1 min). Following dehydration and clearing, slides were mounted with neutral balsam. After staining, slides were examined under a bright-ﬁeld microscope (VS200, OLYMPUS, Japan).

**Cell viability assay**

HUVECs were seeded in 96-well plates at a density of 4 × 10³ cells per well. Cell viability was measured using the Cell Counting Kit-8 (CCK-8; Yeasen Biotech, China). A 100 μL medium containing 10% CCK-8 reagent was added per well. Absorbance at 450 nm was measured using a FLUOstar Omega microplate reader (BMG Labtech, Germany) after 2 hours of incubation.

**Wound-scratch assay**

HUVECs were cultured in a 35-mm plastic dish equipped with an Ibidi Culture-Insert chamber (81176; Ibidi, Gräfelfing, Germany). After the cells reached confluence, the insert was carefully removed to generate a uniform wound. Images were acquired at 0 and 24 h using an inverted bright-field microscope (CKX41, Olympus, Japan). Wound closure was quantified by measuring the residual gap area using ImageJ (NIH, USA).

**Tube formation assay**

Cell well plates were pre-coated with 50 μL Matrigel matrix (Corning, USA) per well and polymerized at 37°C for 30 min. HUVECs were trypsinized and resuspended in endothelial cell medium, and seeded onto the solidified Matrigel at a density of 1×10^4^ cells per well. Cells were incubated under with fresh endothelial cell medium supplemented with Yoda1 or Y27632 for 4–6 h to allow capillary-like network formation. Images were acquired using a brightfield inverted microscope (CKX41, Olympus, Japan). Tube formation was quantified by counting enclosed meshes using ImageJ software with the Angiogenesis Analyzer plugin (NIH, USA).

**m/lEV uptake**

m/lEVs were labeled with the PKH26 Red Fluorescent Cell Linker Kit (Sigma-Aldrich) following the manufacturer’s instructions. Briefly, m/lEVs in 250 μL Diluent C were mixed with 1 μL PKH26 in 250 μL Diluent C. After a 4 min incubation at room temperature, the reaction was terminated with 500 μL 1% BSA in PBS. Unbound dye was removed via differential centrifugation. Splenic capsule SMCs (scSMCs) were then treated with equal amounts of PKH26-labeled EVs. Cellular uptake was visualized using a confocal microscope (LSM 800, Zeiss, Germany).

**Transfection**

The PIEZO1-GFP pEG Bacmam plasmid was generously provided by Prof. Yixiao Zhang from the Chinese Academy of Sciences, expresses a Piezo1-GFP fusion protein and was constructed as described by Zhou Z et al. (Science, 2023). The mPiezo1-GST-IRES-GFP plasmid was generously provided by Prof. Bailong Xiao (Tsinghua University), was constructed as described by Ge J et al. (Nature, 2015). All constructs were verified by Sanger sequencing (Tske Biotechnology, China).

siRNA duplexes were custom-designed and synthesized by Genepharma (China). Cells were seeded in 6-well plate and cultured to 70–80% confluency before transfection. For DNA transfection, 1 μg of plasmid DNA was diluted in 200 μL of Opti-MEM® (Gibco,USA), followed by the addition of 4 μL of X-tremeGENE™ HP Reagent (Roche, USA). For RNA transfection, 4 μg of siRNA was diluted in 100 μL of Opti-MEM® (Gibco, USA). In a separate tube, 6 μL of Lipofectamine™ RNAiMAX (Invitrogen, USA) was diluted in the same volume of Opti-MEM®. The mixture was vortexed gently and incubated (10 min, RT) for complex formation. Subsequently, the complexes were added dropwise to the cells. Cells were harvested or analyzed after 60 h post-transfection for analysis. siRNA sequences are shown in Table S3.

**qPCR**

Total RNA was extracted from the spleen capsule and reverse-transcribed into cDNA. Quantitative real-time PCR was then performed using SYBR Green chemistry, and the relative expression of target genes was normalized to *Actin* and calculated via the 2^(-ΔΔCt) method. Primer sequences are shown in Table S4.

**Immunostaining**

Fresh tissues were embedded in a tissue mold using optimal cutting temperature compound (Sakura Finetek, Japan). Transverse sections were sectioned at 8 μm thickness and stored in the 80˚C freezer before staining. Cells were washed with PBS (Sangon Biotech, China) for three times before staining. Cell or tissue samples were fixed with 4% paraformaldehyde for 10 min. Samples were permeabilized with 0.1% Triton X-100 (Sigma-Aldrich, USA), blocked with goat serum (Gibco, USA) for 1 h and incubated overnight at 4˚C with primary antibodies (1:200 dilution). The next day, samples were incubated with secondary antibodies (1:500 dilution) for 2 hours at room temperature. Immunofluorescence images were acquired using the confocal microscopy (LSM 800; Zeiss, Germany). Antibodies against CD31 (ab7388), CD63 (ab1318), Annexin A1(ab214486), were purchased from Abcam (USA). Antibodies against Piezo1 (15939) were purchased from Proteintech (China). Antibodies against α smooth muscle actin (α-SMA, 48938S) was sourced from CST (USA). Antibodies against tyrosine hydroxylase (TH, 25859) was provided by GeneTex (USA). α1 adrenergic receptor (α1AR, A9410) was purchased from Abclonal (China).

**Western blotting**

Western blotting was carried out as previously described. Protein concentrations were determined using a bicinchoninic acid (BCA) assay kit (#23225; Thermo Fisher Scientific, USA) with bovine serum albumin standards. Proteins were separated by SDS-PAGE, transferred to PVDF membranes, and incubated with target antibodies. Signals were detected using ECL reagent (#32106; Thermo Fisher, USA) and quantified by ImageJ (v1.53; NIH). Antibodies against p-Piezo1 was generously provided by Prof. Bailong Xiao (School of Pharmaceutical Sciences, Tsinghua University). Antibodies against CD63 (ab1318), Annexin A1(ab214486), were purchased from Abcam (USA). Antibodies against Piezo1 (15939), Lamin B (12987), ATPase Na+/K+ transporting subunit alpha 1 (ATP1A1, 14418), tumor susceptibility gene 101 protein (TSG101, 28283), Tranfsferrin (PA00119), ALG-2 interacting protein X (ALIX, 12422), Calnexin (10427), apolipoprotein B (APOB, 840047), CD9 (20597), asialoglycoprotein receptor (ASGPR, 83691), CD31 (111265), Albumin (16475), GFP (50430) and Tubulin (66031) were purchased from Proteintech (China). Antibodies against endothelial nitrate oxide synthase (eNOS, 32027s), phospho-eNOS (p-eNOS, 9570), cleaved caspase 3 (9661) were sourced from CST (USA). CD9 (A1703), matrix metallopeptidase 2 (MMP2, A23496), myosin light chain 2 (MLC, A14188), p-MLC (AP0955), vascular endothelial growth factor receptor (VEGFR2, A5609), p-VEGFR2 (AP0382), protein kinase C (PKC, A17922), p-PKC (AP0560), Yes-associated protein (Yap, A1001) and p-Yap (AP0489) were purchased from Abclonal (China).

**Drugs and chemical compounds**

Drugs were obtained from Med Chem Express (USA). Phenylephrine (PE, HY-B0769), norepinephrine (NE, HY-13715), Y27632 (HY-10071), and midodrine hydrochloride (HY-12749) were dissolved in H_2_O for *in vitro* studies. Thapsigargin (TG, HY-13433), BAPTA (HY-100168), Gö 6976 (HY-10183), GsMTX4 (HY-P1410), Methoxamine hydrochloride (HY-B1298), PD150606 (HY-100529), KN-62 (HY-13290), Prazosin (HY-B0193), A23187 ([HY-N6687](https://www.medchemexpress.cn/calcimycin.html)) and Yoda1 (HY-18723) were dissolved in DMSO (HY-Y0320C) for *in vitro* studies. Oxidopamine hydrobromide (6-OHDA, HY-B1081A), Pantethine ([HY-B1028](https://www.medchemexpress.cn/Pantethine.html)) and Y27632 (HY-10071) were dissolved in sterile saline solution for *in vivo* studies. Dynasore (HY-15304) was dissolved in 10% DMSO and 90% (20% [SBE-β-CD](https://www.medchemexpress.cn/SBE-_beta_-CD.html) [HY-17031] in saline) for *in vivo* studies. For Yoda1 *in vivo* studies, the Yoda1 stock solution was further diluted in corn oil to achieve a final composition of 10% DMSO and 90% corn oil (HY-Y1888) (v/v). Tamoxifen (HY-13757A) was dissolved in corn oil.

**Supplementary figures and figure legends**

**Figure S1. The filtration step during extracellular vesicle (EV) isolation shows no significant effect on medium/large EVs (m/lEVs) morphology and function**

(A)The size distribution of m/lEVs remained unchanged following the filtration step (n = 3). (B) Piezo1 and Annexin A1 protein levels were not altered by the filtration (n = 4). (C) The morphology of m/lEVs was preserved after the filtration step (n = 4). (D) Filtration did not affect the ability of m/lEVs to stimulate splenic capsule smooth muscle cell (scSMC) contraction (n = 6). Data are presented as mean ± SEM. ***P*<0.01.

**Figure S2. Characterization of laminar shear stress (LSS)-induced m/lEVs**

(A) Cell viability remained unchanged following the short-term LSS stimulation (n = 5). (B) LSS did not affect size distribution of the m/EVs as detected by DLS (n = 3). (C) Activation of Piezo1 promoted EV release in HAECs, VSMCs, A549 cells and HepG2 cells (n = 20). (D) The size distribution of m/lEVs in HAECs, VSMCs, A549 cells and HepG2 cells (n = 3). Data are presented as mean ± SEM. ***P*<0.01.

**Figure S3. Quantitative analysis of protein levels for Figure 2**

(A) Quantification of protein levels in Figure 2E and 2F (n = 3). (B) Quantification of protein levels in Figure 2I and 2J (n = 3). (C) Quantification of protein levels in Figure 2M (n = 3). (D) Quantification of protein levels in Figure 2P (n = 3). Data are presented as mean ± SEM. ***P*<0.01.

**Figure S4. Blocking m/lEV release attenuates exercise- or Yoda1-induced endothelial benefits**

(A-C) Administration of the microvesicle inhibitor pantethine (50 μM) or Y27632 (2 μM) reduced Yoda1 (Piezo1 agonist, 2 μM)-induced benefits in tube formation (A), ACh-induced NO production (B), and VEGFR2 and eNOS phosphorylation (C) (n = 5 for A-B and n = 3 for C). (D) Exercise increased the levels of EV markers in plasma-derived m/lEVs (n = 8). (E) Both exercise and Y27632 (5 mg/kg, i.p.) injection decreased mouse body weight, pantethine (150 mg/kg, i.p.) showed no impact on body weight (n = 8). (F) Coordination quantiﬁed from average latency to fall (n = 8). (G) Both pantethine and Y27632 inhibited the effect of exercise on increasing eNOS phosphorylation in aorta (n = 5). (H) Pantethine and Y27632 inhibited the effect of exercise on upregulating circulating NO (n = 8). (I) One-month exercise combined with pantethine or Y27632 increased aortic pulse wave velocity (PWV) (n = 8). Data are presented as mean ± SEM. ***P*<0.01.

**Figure S5. Piezo1 peripheral translocation is necessary for packaging Piezo1 into m/lEVs**

(A) Both Yoda1 and A23187 induced m/lEV release (n = 100 cells from 3 independent experiments). (B) Yoda1 promoted the translocation of Piezo1 to the cell periphery, whereas A23187 did not (n = 3). (C) Confocal microscope visualized that Yoda1 increased the content of Piezo1 in m/lEVs, whereas no such increase was observed following A23187 treatment. (D) Western blot analysis showed that Yoda1 but not A23187 increased Piezo1 content in isolated m/lEVs (n = 4). Data are presented as mean ± SEM. **P*<0.05, ***P*<0.01.

**Figure S6. Piezo1 was observed in Yoda1-induced m/lEVs in other types of cells**

**Figure S7.** **Endothelium is a primary source of exercise-induced circulating m/lEVs.**

(A) Representative images of Piezo1 (green), α-SMA (red; smooth muscle cell [SMC] marker), CD31 (red; endothelial cell [EC] marker) and DAPI (blue, nuclear staining) in *en face* preparations of aortas from Piezo1^fl/fl^, Piezo1_SMC_^-/-^ and Piezo1_EC_^-/-^ mice. (n = 3). (B) Upper panel: Schematic of genetically engineered mouse model construction. Lower panel: Piezo1 expression in the liver of Piezo1_Hep_^-/-^ mice (n =6 ). (C) Acute exercise-induced upregulation of Piezo1, Annexin A1, CD9 and MMP2 in circulating m/lEVs was unchanged in Piezo1_Hep_^-/-^ mice (n =6). (D) Exercise-induced m/lEVs exhibited increased levels of the endothelial marker CD31, with no significant change in the hepatocyte marker ASGPR (n = 6). (E) Exercise did not increase the mRNA level of Piezo1 in the liver and spleen (n = 6). (F) m/lEVs derived from ECs isolated from Piezo1_EC_^-/-^ mice did not contain Piezo1 protein (n = 6). (G) Isolated EVs from ECs with overexpression of Piezo1-GFP contain Piezo1-GFP. Data are presented as mean ± SEM. ***P*<0.01.

**Figure S8. Piezo1 redistribution to the spleen promotes spleen contraction**

(A) Dynasore (a dynamin-dependent endocytosis inhibitor, 50 μM) attenuated the uptake of HUVEC-derived m/lEVs by scSMCs (n = 5). (B) Inhibition of Piezo1 uptake through dynasore (10 mg/kg, i.p.) reduced the effect of exercise on upregulating splenic Piezo1 contents (n = 6). (C) Dynasore attenuated exercise-induced spleen contraction (n = 10). Upper panel: Representative ultrasound images of the spleen pre- and post-exercise. Lower left panel: Quantification of spleen area pre- and post-exercise from ultrasound images. Lower right panel: Percentage change in spleen contraction in response to exercise. Ultrasonographic measurements were performed pre- and post-exercise. (D) EC-specific KO (Piezo1_EC_^-/-^), Y27632 (5 mg/kg, i.p., 30 min prior to NE injection) and GsMTx4 (10 mg/kg, i.p., 30 min prior to NE injection) reduced NE (adrenergic receptors agonist, 1mg/kg, i.p.)-induced spleen contraction (n = 10). Ultrasonographic measurements were performed before and at 1 h after all drugs injection. (E) 6-OHDA (chemical denervation, 100 mg/kg, i.p., 24 h prior to exercise) abolished exercise-induced spleen contraction (n = 10). Ultrasonographic measurements were performed pre- and post-exercise. (F) Yoda1 (2 mg/kg, i.p.) still induced spleen contraction even with chemical denervation by 6-OHDA (100 mg/kg, i.p., 24 h prior to Yoda1 injection) (n = 10). Ultrasonographic measurements were performed before and at 1 h after Yoda1 injection. Data are presented as mean or mean ± SEM. **P* < 0.05, ***P* < 0.01.

**Figure S9. Systemic hemodynamic responses to exercise and identification of dominant factors driving spleen contraction.**

(A) A single bout of exercise increased heat rate (HR) and cardiac output (CO) (n = 20). (B) Acute exercise promoted popliteal artery blood flow (n = 20). (C) Feature importance ranking in the Lasso model for predicting coronal plane spleen contraction magnitude. Bar length represents contribution weight (n = 20). (D) Feature importance ranking for transverse plane spleen contraction prediction (n = 20). Data are presented as mean in the bar graph. Data are presented as the median with interquartile range in the violin plots. ***P* < 0.01.

**Supplementary tables**

**Table S1. Volunteer characteristics**

|  | All participants  (n = 20) | Males  (n = 13) | Females  (n = 7) |
| --- | --- | --- | --- |
| Age, mean (SD), y | 27.60 (3.50) | 26.23 (2.24) | 30.14 (4.14) |
| Height, mean (SD), cm | 172.37 (9.69) | 177.65 (6.90) | 162.57 (5.44) |
| Weight, mean (SD), kg | 70.45 (12.78) | 75.35 (12.43) | 61.36 (7.76) |
| BMI, mean (SD), kg/m^2^ | 23.60 (3.00) | 23.75 (2.73) | 23.31 (3.67) |
| Heart rate, mean (SD), bpm | 68.15 (6.56) | 66.92 (6.89) | 70.43 (5.68) |

**Table S2. Volunteer characteristics and ultrasonographic profiles**

| ID | Spleen contraction  (Coronal plane) （%） | Spleen contraction  (Transverse plane) （%） | CD9  (Post/Pre) | Piezo1 (Post/Pre) | CO (Post/Pre) | Heart rate (Post/Pre) | HRmax (bpm) | Popliteal artery blood flow (post/pre) | Height (cm) | Weight (kg) | Age (y) | Sex |
| --- | --- | --- | --- | --- | --- | --- | --- | --- | --- | --- | --- | --- |
| 1 | 21.83 | 2.10 | 1.12 | 0.57 | 1.33 | 2.48 | 160.00 | 1.15 | 180.00 | 77.50 | 25 | male |
| 2 | 34.79 | 39.85 | 1.79 | 0.96 | 1.45 | 3.05 | 191.00 | 2.17 | 155.00 | 69.00 | 29 | female |
| 3 | 26.86 | 55.83 | 1.80 | 2.59 | 1.31 | 2.67 | 184.00 | 3.06 | 170.00 | 71.00 | 29 | male |
| 4 | 47.18 | 40.75 | 2.41 | 2.44 | 1.11 | 3.23 | 181.00 | 3.13 | 181.00 | 90.50 | 25 | male |
| 5 | 22.69 | 33.02 | 1.89 | 2.62 | 1.73 | 2.77 | 177.00 | 2.23 | 190.00 | 95.00 | 30 | male |
| 6 | 7.33 | 6.74 | 0.72 | 1.49 | 1.83 | 1.93 | 162.00 | 1.01 | 183.00 | 87.00 | 24 | male |
| 7 | 15.78 | 21.23 | 1.14 | 1.04 | 1.30 | 2.80 | 168.00 | 1.42 | 178.00 | 80.00 | 28 | male |
| 8 | 39.49 | 12.00 | 1.42 | 1.26 | 2.24 | 2.87 | 178.00 | 2.13 | 165.00 | 64.00 | 28 | male |
| 9 | 14.25 | 3.52 | 1.74 | 1.03 | 2.29 | 2.34 | 164.00 | 1.77 | 161.00 | 74.00 | 39 | female |
| 10 | 33.09 | 55.15 | 1.52 | 1.47 | 1.37 | 2.62 | 173.00 | 3.78 | 163.00 | 55.00 | 29 | female |
| 11 | 26.66 | 7.80 | 1.14 | 0.77 | 2.00 | 2.31 | 169.00 | 1.88 | 175.00 | 65.00 | 27 | male |
| 12 | 26.80 | 17.02 | 1.88 | 1.06 | 2.03 | 2.44 | 178.00 | 2.33 | 180.00 | 88.00 | 26 | male |
| 13 | 44.64 | 32.07 | 1.81 | 2.77 | 2.34 | 2.85 | 177.00 | 2.18 | 186.00 | 79.00 | 23 | male |
| 14 | 26.75 | 65.36 | 2.08 | 1.33 | 1.25 | 2.54 | 173.00 | 1.92 | 177.50 | 63.50 | 24 | male |
| 15 | 27.56 | 14.21 | 2.08 | 1.37 | 1.69 | 2.45 | 173.00 | 1.19 | 165.00 | 57.50 | 27 | female |
| 16 | 22.23 | 16.03 | 1.29 | 1.52 | 1.30 | 2.40 | 186.00 | 2.16 | 172.00 | 64.00 | 29 | female |
| 17 | 10.77 | 24.50 | 0.73 | 1.03 | 0.91 | 2.44 | 183.00 | 1.73 | 158.00 | 55.00 | 27 | female |
| 18 | 35.51 | 37.84 | 1.56 | 2.50 | 1.35 | 2.59 | 189.00 | 1.70 | 170.00 | 61.00 | 28 | male |
| 19 | 28.82 | 14.29 | 1.82 | 1.53 | 0.89 | 2.22 | 173.00 | 2.03 | 164.00 | 55.00 | 31 | female |
| 20 | 36.36 | 34.32 | 2.17 | 3.05 | 2.04 | 2.44 | 173.00 | 1.59 | 174.00 | 58.00 | 24 | male |

**Table S3. siRNA sequences**

| Target | Sense strand (5’-3’) | Antisense strand (5’-3’) |
| --- | --- | --- |
| si-*Piezo1*-1 | GGUCCUACCUUGACAUGCUTT | AGCAUGUCAAGGUAGGACCTT |
| si-*Piezo1*-2 | CCUCAAGAGCAUUGACUUUTT | AAAGUCAAUGCUCUUGAGGTT |
| si-*Mlc*-1 | GGAAACCAUUCUCAACGCATT | UGCGUUGAGAAUGGUUUCCTT |
| si-*Mlc*-2 | GGGUCCAAUUAACUUUACUTT | AGUAAAGUUAAUUGGACCCTT |
| Scramble | UUCUCCGAACGUGUCACGUTT | ACGUGACACGUUCGGAGAATT |

**Table S4. Primer sequences for mRNAs**

| Gene | Forward primer (5’-3’) | Reverse primer (5’-3’) |
| --- | --- | --- |
| *Piezo1* | GGAAAAGAGCTCCGACACAC | CCAGGACTTCCCCACCTATT |
| *Actin* | GTTGGAAGACCGAGGTTTACTA | ACAATGCCTTCTTCCTGTAGTT |
